# Hunter-Gatherers and the Origins of Religion

**DOI:** 10.1007/s12110-016-9260-0

**Published:** 2016-05-06

**Authors:** Hervey C. Peoples, Pavel Duda, Frank W. Marlowe

**Affiliations:** Department of Archaeology and Anthropology, University of Cambridge, Cambridge, UK; P.O. Box 8850, Longboat Key, FL 34228 USA; Department of Zoology, University of South Bohemia, České Budějovice, Czech Republic

**Keywords:** Religion, Evolution, Hunter-gatherers, Animism, High gods, Cultural phylogenetics

## Abstract

Recent studies of the evolution of religion have revealed the cognitive underpinnings of belief in supernatural agents, the role of ritual in promoting cooperation, and the contribution of morally punishing high gods to the growth and stabilization of human society. The universality of religion across human society points to a deep evolutionary past. However, specific traits of nascent religiosity, and the sequence in which they emerged, have remained unknown. Here we reconstruct the evolution of religious beliefs and behaviors in early modern humans using a global sample of hunter-gatherers and seven traits describing hunter-gatherer religiosity: animism, belief in an afterlife, shamanism, ancestor worship, high gods, and worship of ancestors or high gods who are active in human affairs. We reconstruct ancestral character states using a time-calibrated supertree based on published phylogenetic trees and linguistic classification and then test for correlated evolution between the characters and for the direction of cultural change. Results indicate that the oldest trait of religion, present in the most recent common ancestor of present-day hunter-gatherers, was animism, in agreement with long-standing beliefs about the fundamental role of this trait. Belief in an afterlife emerged, followed by shamanism and ancestor worship. Ancestor spirits or high gods who are active in human affairs were absent in early humans, suggesting a deep history for the egalitarian nature of hunter-gatherer societies. There is a significant positive relationship between most characters investigated, but the trait “high gods” stands apart, suggesting that belief in a single creator deity can emerge in a society regardless of other aspects of its religion.

**Electronic supplementary material:**

The online version of this article (doi:10.1007/s12110-016-9260-0) contains supplementary material, which is available to authorized users.

Religion is unique to humans. Belief in supernatural agents and the entailed religious practices occur in virtually all human cultures (Brown 1991; Johnson 2005; Murdock 1965; Murdock and White 1980). The universality of religion across human societies (Brown 1991) suggests a deep evolutionary past. The human ability to create complex culture (“cultural capacity”) may be older than the first split in the modern human lineage (Lind et al. 2013), and some aspects of religion may have been present before the appearance of anatomically modern humans (Rossano 2006). Religion has generally been assumed to have emerged among anatomically modern humans in Africa during the Upper Paleolithic, and to have played a vital role in the subsequent out-of-Africa expansion (Balme et al. 2009; Rossano 2009a).

Explanations for a natural emergence of religion have been debated for hundreds, if not thousands, of years (Martin and Wiebe 2012; Wiebe 2008). That debate has now turned to empirical approaches and testable hypotheses, many of them grounded in the framework of evolutionary theory (Alcorta and Sosis 2005; Atran and Norenzayan 2004; Barrett and Lanman 2008; Boyer and Bergstrom 2008; Irons 2001; Rossano 2006; Tremlin 2006). During the past decade, evolutionary psychologists have identified and described the activity of cognitive biases that enable us to accept the counterintuitive concepts and beliefs of religion (Atran and Henrich 2010; Barrett and Lanman 2008; Tremlin 2006). Research into the dynamics of religion has revealed the nature of ritual behavior to promote high levels of cooperation (Atran and Henrich 2010; Fischer et al. 2013; Sosis and Ruffle 2003; Xygalatas et al. 2013). The contribution of belief in morally punishing high gods to enhancing prosociality and ensuring growth and stabilization of society has also been demonstrated (Norenzayan and Shariff 2008; Norenzayan et al. 2016; Peoples and Marlowe 2012).

This research has often focused on characteristics of the large prosocial religions that have emerged during the 10,000–12,000 years since the advent of agriculture (Matthews 2012; Norenzayan 2013). Little attention has been paid to the religion of hunter-gatherers whose religious beliefs and behaviors have been evolving during the vast majority of human history (Lee and DeVore 1968). Despite established speculations about various beliefs and behaviors that may represent an original form of religion, specific traits of nascent religiosity, and the sequence in which they emerged, have remained unknown.

There have been as many attempts to define religion as to explain its origins. Broadly defined, religion is a set of beliefs and behaviors based on a shared worldview that separates the sacred, or supernatural, from the profane (Durkheim 1965 [1912]). In this study of its origins, we view religion as a biocultural adaptation (Alcorta and Sosis 2005; Harris and McNamara 2008; Sanderson and Roberts 2008).

How old are religious concepts and their engendered behaviors? Ritual behavior is widespread among humans today, operating in a variety of social environments, both religious and secular (Brown 1991; Cronk 2005; Spencer 1870). Behavior reminiscent of ritual can be seen in many animals, including the ecstatic “rain dances” and directional drumming of chimpanzees (Goodall 1986; Nishida et al. 1999), which along with bonobos are our closest extant evolutionary kin. It is highly likely that archaic hominins would have exhibited ritualistic behavior in some form, but evidence for nascent religiosity remains difficult to infer from the archaeological record (Rossano 2006, 2009b).

Whether early hominins held religious beliefs prior to the emergence of language is unknown. We should not dismiss the possible presence of non-linguistic religious thought and sentiment among early members of the genus *Homo*. However, a case can be made that transmission of religious concepts from one individual to another requires complex mental imaging, and a capacity for symbolic thought and communication that might include ritual, dancing, singing, gestures, art and ornamentation, as well as language (Deacon and Cashman 2010; Mithen 1998). Some genetic and anatomical changes enabling speech and language can be traced to the most recent common ancestor of Neanderthals and anatomically modern humans about half a million years ago (Barney et al. 2012; Dediu and Levinson 2013; Krause et al. 2007). It has been suggested that rudimentary language could be older than symbolic thought (Barnard 2012).

The archaeological record documents the presence of artifacts and technology dating from ca. 400 kya that would probably have required a level of symbolic communication close to that of language (Zilhão 2007). Early evidence for the processing and use of red ochre, often considered a marker for symbolic behavior, dates to the Middle Pleistocene >400 kya in Africa (Barham 2002) and to >284 kya (Deino and McBrearty 2002) in the presence of Levallois blade technology.

Finds at Pinnacle Point in southern Africa (Marean et al. 2007) demonstrate the use and processing of pigment among anatomically modern humans as early as 165,000 years ago (McDougall et al. 2005). Ochre nodules bearing engraved abstract patterns and perforated shell beads found at Blombos Cave in South Africa, dating to 75,000–100,000 years ago (d’Errico et al. 2005; Henshilwood et al. 2009), suggest symbolically mediated behavior. These and other similar finds lend substantial support to the theory of progressive development of symbolic behavior and complex imagery along with the evolution of modern human anatomy (Barnard 2012; Conard 2010; Deino and McBrearty 2002; d’Errico and Stringer 2011; McBrearty and Brooks 2000; Zilhão 2007). Pleistocene hunter-gatherers would most likely have possessed both the cognitive and communicative skills to share religious beliefs and practices prior to dispersal out of Africa more than 60 kya (Fu et al. 2013; Henn et al. 2012; Johansson 2011; Lind et al. 2013). Although present-day hunter-gatherers are not direct analogues of those early societies and may not be direct, unbroken descendants of ancestral hunter-gatherers, they can provide a window onto traits selected for in the Pleistocene (Marlowe 2005), including traits of early religion.

The uniqueness of “natural” religions of hunter-gatherers, and likely those of our Paleolithic ancestors, cannot be overemphasized when compared with the “world” religions that have emerged along with the advent of agriculture. Many hunter-gatherer societies have little or no concept of religion per se, though a religious dimension often permeates normal activities and is continuous with daily life (Lee 1989). Hunter-gatherer religions are seldom religions of protest or evangelism (Woodburn 1997). Instead, each society focuses on maintaining its unique beliefs and culture, along with a sense of self-worth and the general health and well-being of the group (Woodburn 1997, 2005). Simple egalitarian hunter-gatherer groups generally hold fewer religious beliefs and participate in less ritual (Marlowe 2010) than more complex groups. But hunter-gatherers do have religion, embodied in sacred healing dances and rituals marking life events. Although there is considerable variation in specific religious traits among hunter-gatherer societies, a cross-cultural view reveals underlying similarities in cosmology, ritual, and belief (Rossano 2007). These often include gods and spirits with limited powers who are typically *not* omniscient and usually lack concern for morality and human affairs (Marlowe 2010; Norenzayan et al. 2016; Peoples and Marlowe 2012; Swanson 1960; Woodburn 1997), as Marshall describes:

The concept of sin as an offence against the gods is vague among the !Kung. Man’s wrong-doing against man is not left to #Gao!na’s punishment nor is it considered to be his concern. Man corrects or avenges such wrong-doings himself in his social context. (Marshall 1962:245)

What were the specific traits of early religion? How did traits of nascent religiosity evolve and interact over time? Recently phylogenetic comparative methods have been increasingly applied to the study of the evolution of material and non-material culture (Mace and Holden 2005; Mace and Jordan 2011), including religion (Matthews 2012; Watts et al. 2015). Reconstructing ancestral character states on phylogenies based on genetic or linguistic data has proven valuable in revealing the history of various sociocultural phenomena (Currie et al. 2010; Opie et al. 2014; Walker et al. 2011, 2012). Although religious beliefs are regarded as one of those cultural traits that are historically labile and prone to cultural borrowing (Guglielmino et al. 1995), cross-cultural research suggests that religion (and mythology) can be surprisingly stable across time and space, and shared religious beliefs can be indicative of deep ancestry (Berezkin 2008; Blust 2013). The use of phylogenetic methods is important for understanding not only the origins of religious traits, but also the behavioral systems that emerged from them that have determined patterns of social constraint and have impacted believers and non-believers alike.

Importantly, homology is not limited to morphology and its genetic and/or developmental underpinnings. Behavior, which is often evolutionarily conserved, is also a proper subject of homology relations and can be used in phylogenetic reconstruction (Hall 2013; Powell and Shea 2014; Rendall and Di Fiore 2007). A behavioral homology need not have a particular structural basis. We do not argue for homology of the particular religious beliefs (e.g., different afterlife beliefs across hunter-gatherer societies that possess this trait) but for the homology of the fundamental religious concepts (e.g., the concept of afterlife itself) and their continuity. Even characters that cannot be hypothesized as strictly homologous among sampled cultures can be analyzed because they can represent non-homologous psychological-behavioral responses to identical selective pressures (see Murdock 1965 for similar reasoning in anthropology).

In this study we investigate early evolution of religion by reconstructing ancestral states for seven characters describing religious beliefs and behaviors in a global sample of 33 hunter-gatherer societies (Fig. 1). Using a time-calibrated supertree based on published genetic and linguistic phylogenetic trees, and linguistic classification as a proxy for population history, we reconstruct ancestral character states and test for correlated evolution between the characters and for the direction of cultural change.

**Fig. 1:**
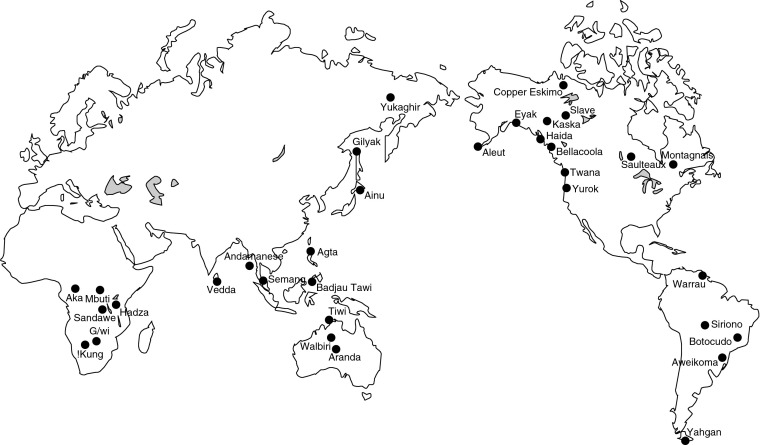
Geographic locations of the 33 hunter-gatherer societies in the study sample

## Methods

Phylogenetic reconstruction of ancestral character states is a two-step process. It requires a set of characters (data matrix) with known character states (e.g., present or absent) based on traits of interest, and a phylogenetic tree that represents the historical relationships between the populations under investigation.

### Population Sample

We used primary ethnographic sources to create a data matrix of characters describing religiosity in a sample of 33 hunter-gatherer societies. The study sample consists of 28 hunter-gatherer societies selected from the Standard Cross-Cultural Sample (SCCS) (Murdock and White 1980), and five additional hunter-gatherer societies selected from both the *Ethnographic Atlas* (EA) (Murdock 1967) and original ethnographic descriptions of the societies.

We used ratings of other researchers to obtain a subsample of 28 hunter-gatherer societies from the SCCS. The variables used to define the sample were SCCS variable 1: Intercommunity Trade as Food Source; variable 3: Agriculture Contribution to Local Food Supply; variable 5: Animal Husbandry Contribution to Food Supply (Murdock and Morrow 1970); and variable 858: Subsistence Type–Ecological Classification (coded by D. White after Paige and Paige 1981). We defined our sample of hunter-gatherers as follows: contribution to local food supply less than 10% agriculture (SCCS v3), less than 10% animal husbandry (SCCS v5), and trade accounting for less than 50% and no more than any single local source (SCCS v1). We excluded mounted hunters (SCCS v858 = 5, mounted hunting) to more accurately represent pre-agricultural hunter-gatherer societies. We excluded eight SCCS hunter-gatherer societies from the sample either because of the lack of phylogenetic information (Ingalik, Micmac, E. Pomo, Yokuts [Lake], Paiute North, Klamath, and Kutenai) or because their main source of subsistence stated in the EA does not classify them as hunter-gatherers (Shavante depend 16–25% on agriculture). We added five additional societies chosen from the EA and original ethnographic sources in order to balance the geographic distribution of the sample. These additional societies are the Aka and G/wi (Africa), Agta (Philippines) and Walbiri (Australia) hunter-gatherers, and the Sandawe (Africa), who are now mainly herders and farmers but share a deep genetic and historical relationship with the Hadza (Tishkoff et al. 2007). Figure 1 shows the geographic locations of the 33 hunter-gatherer societies in the study.

### Trait Definition and Character Matrix Construction

Original coding of data in all 33 sample societies for the traits of animism (Tylor 1871), belief in an afterlife (Bering 2006), shamanism (Eliade 1964; Winkelman 1990), and ancestor worship (Sheils 1975; Spencer 1870; Steadman et al. 1996; Swanson1960) was based on principal ethnographic source descriptions (White 1989) and additional ethnologies referenced in the character matrix.

Tylor defined animism as a general belief in the “animation of all nature” (1871:258) and fundamental to religion. Animism includes a “belief in personal souls” (1871:260) as well as “a sense of spiritual beings. . . inhabiting trees and rocks and waterfalls” (1871:260). We define animism as the belief that all “natural” things, such as plants, animals, and even such phenomena as thunder, have intentionality (or a vital force) and can have influence on human lives. Animism is coded as present or absent in each society, based on assessments of principal ethnographers. Belief in an afterlife is defined as belief in survival of the individual personality beyond death (Bering 2006) and is coded as either present or absent.

A global definition of shamanism remains contentious (Sidky 2010). We define shamanism as the presence in a society of a “shaman” (male or female), a socially recognized part-time ritual intercessor, healer, and problem solver (Sidky 2010; Winkelman 1990). Shamans often use their power over spirit helpers during performances involving altered states of consciousness (Eliade 1964; Winkelman 2010) to benefit individuals and the group as a whole (Eliade 1964; Winkelman 1990, 2010). We view shamans as a general category of individuals often found in hunter-gatherer societies who mediate between the earthly and spirit worlds to promote cohesion and physical and mental well-being in the society (Eliade 1964; Sidky 2010; Winkelman 1990, 2010). Shamanism is coded as present or absent.

Ancestor worship is defined as belief that the spirits of dead kin remain active in another realm where they may influence the living, and can be influenced by the living (Sheils 1975; Spencer 1870; Steadman et al. 1996; Swanson 1960). Ancestor worship is coded as four states in the SCCS: a belief in ancestor spirits can be (1) absent in a society or the spirits can be (2) present but inactive in human affairs. In other societies, ancestor spirits are believed to be active in human affairs but (3) may not or (4) may be influenced by humans through prayer and sacrifice (Sheils 1975; Spencer 1870; Steadman et al. 1996; Swanson 1960). “High gods” is EA variable 34 and SCCS variable 238, defined by Swanson (1960) as single, all-powerful creator deities who may be active in human affairs and supportive of human morality. The variable is coded as four states. It differentiates between societies in which a creator deity is (1) absent, (2) present but inactive in human affairs, (3) active in human affairs but does not support a moral agenda, or (4) active and morally punishing. The SCCS provides coding for high gods in 28 of the 33 societies in our sample. Original coding in the additional five societies, based on principal ethnographic sources, completed the coding for all 33 societies.

Based on the five traits of hunter-gatherer religion described above, we created a set of seven characters of hunter-gatherer religiosity. The character matrix was coded as follows: animism (absent, present); belief in an afterlife (absent, present); shamanism (absent, present); ancestor worship (absent, present); high gods (absent, present). Two additional traits, active ancestor worship (absent, present) and active high gods (absent, present), were derived from the basic traits of ancestor worship and high gods. These two additional characters recognize those societies that not only hold a belief in ancestor spirits or high gods but also believe that those spirits or high gods are active in human affairs. “Active ancestor worship” denotes the absence or presence of ancestral spirits actively meddling in human affairs, who may or may not be influenced through prayer or sacrifice (Sheils 1975; Spencer 1870; Steadman et al. 1996; Swanson 1960). Similarly, “active high gods” identifies societies that believe in a high god that is also *active* in human affairs, and may or may not be morally punishing (Swanson 1960).

**Fig. 2:**
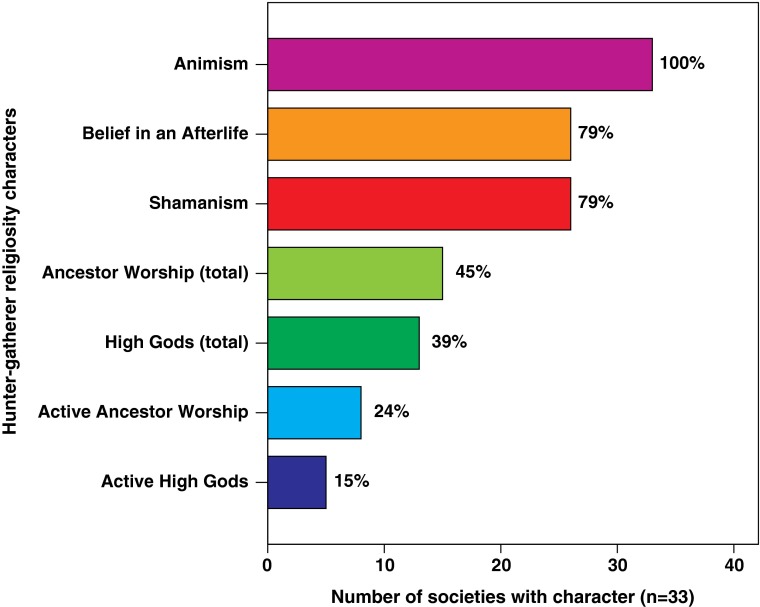
Distribution of the seven characters describing hunter-gatherer religiosity in the study sample

The resulting character matrix has 33 terminals (hunter-gatherer populations) and 7 characters (Fig. 2). (Character matrix and coding references are provided in the ESM, Tables A1a and b.)

### Phylogenetic Supertree Inference

We used a dataset described in Duda and Zrzavý (2016) to generate a phylogeny of 33 hunter-gatherer populations using the Matrix Representation with Parsimony (hereinafter MRP) supertree method (Baum 1992; Ragan 1992). The dataset is based on 301 genetic and linguistic source trees from 199 studies published in journals, edited volumes, and books from 1990 to ca. 2014. It also includes a character set based on language classifications of Greenberg (1987), Ruhlen (1991), and Greenberg and Ruhlen (2007) on the level of linguistic stocks and families.

Data from White (2009) and *Ethnologue* (Lewis et al. 2013) were used to match the hunter-gatherer populations in the study sample with the populations present in the supertree dataset.

Populations absent from or underrepresented in the supertree dataset were replaced by either a more inclusive population group or by a genetically closely related population that was used as a proxy for the hunter-gatherer population in question. Positions of four North American populations (Kaska, Eyak, Twana, and Yurok) were based solely on linguistic classification (ESM Table A2).

In order to overcome the problem of the lack of genetic data and the conflicting signals caused by recent genetic admixture and language shifts in some hunter-gatherer populations in the study sample, the characters based on linguistic classifications were up-weighted by a factor of 100 to serve as a topological constraint or “scaffold.” This scaffold tree constrains the topology for a subset of populations for which linguistic affiliation can be determined (i.e., those scored for characters). (Details on supertree dataset manipulations are given in the Methods section of the ESM.)

The linguistic scaffold tree included 20 phylogenetically informative characters for the 33 populations in the study sample. Note that this linguistic scaffold implies relatively few internal groupings (clades based on linguistic classification), particularly among the Old World hunter-gatherers (see ESM Figure A1).

Semi-rooted coding (sensu Bininda-Emonds et al. 2005) was employed. The supertree was rooted by an “all-0” hypothetical outgroup that preserves the rooting information for rooted source trees; for unrooted source trees the hypothetical outgroup was scored as “?”.

The complete dataset included 974 taxa + outgroup. The analysis was performed in TNT 1.1 (Goloboff et al. 2008) under “new technology search” with search level 10 using sectorial, ratchet, and tree fusing searches, obtaining trees from a 10,000-replicate random addition sequence, treating gaps as missing data and all character changes as equal and non-additive. The recovered trees were then subjected to additional branch swapping with up to 10,000 trees held during each step. The resulting supertree is a semi-strict consensus tree reduced to 33 hunter-gatherer populations + outgroup. The topology of this supertree is fully resolved (ESM Figure A2).

### Time-Calibrating the Supertree

Time-calibrated branch lengths were obtained from published time estimates of divergence events and colonization events in human population history based on molecular, linguistic, and archaeological data (ESM Table A3b). These estimates were used as time constraints on the nodal ages of the supertree. In order to test the robustness of reconstructions of ancestral character states and given the considerable variance in molecular-based time estimates of divergence dates and discrepancies between estimates based on molecular, linguistic, and archaeological data, two sets of time estimates were used. (Two sets of divergence dates for time-calibrating the supertree are given in ESM Table A3a.)

The first set of dates assumes a greater time depth of the supertree and consists mostly of molecular-based estimates of divergence dates. The second set of divergence dates is based on molecular-based estimates, archaeological data, and glottochronology and is close to the minimum time estimates. We emphasize the results based on the second set of dates since these represent the time when the populations in our sample last shared close cultural contact, which arguably suits our analyses better than the estimates of deeper, molecular-based divergence dates between the populations in question.

The age of the nodes for which time estimates were available was fixed using the Node Age Constraint tool in Mesquite 3.02 (Maddison and Maddison 2015). The time-calibrated supertree was inferred using the combination of Enforce Minimum Node Age Constraints and Arbitrarily Ultrametricize functions in Mesquite 3.02 (Maddison and Maddison 2015).

### Phylogenetic Reconstruction of Ancestral Character States

The set of characters was mapped onto the tree topology. All character states in the outgroup were scored as 0, absent (i.e., plesiomorphic character states for all characters). Maximum parsimony and maximum likelihood reconstruction of ancestral character states were performed in Mesquite 3.02 (Maddison and Maddison 2015). The Markov k-state 1 parameter model (Mk1) that assumes an equal rate of change between all character states (Lewis 2001) was used for maximum likelihood reconstruction. An asymmetric likelihood ratio test (Pagel 1999b) indicated that the asymmetric two-parameter model does not offer a significant improvement over the Mk1 model for any of the seven characters in question, thus validating the Mk1 model. Each character was mapped onto a set of topologies using the Trace Character History function.

The statistical support for the ancestral state reconstructions was determined using a likelihood decision threshold of *T* = 2, indicating support at least 7.4 times greater for the character state in question than for the alternative character state(s) (Schluter et al. 1997).

### Testing for Correlated Character Evolution

In order to test hypotheses about temporal ordering of character state changes and coevolution of traits we used Pagel’s test for correlated discrete character evolution (Pagel 1994, 1999a) implemented in the Pagel94 module in Mesquite 3.02 (Maddison and Maddison 2015). This method uses a continuous-time Markov model to infer character changes along each branch of a phylogenetic tree in order to establish the most likely temporal ordering and direction of evolutionary change and the most probable evolutionary pathway between two discrete binary characters. Evolutionary change in each character along the tree branches is modelled as a Markov process, in which the likelihood of character change is dependent on its current character state. Two models are fitted: an independent, four-parameter model (L_i_) in which evolution in each character is independent of the state of the other character, and a dependent, eight-parameter model (L_d_) in which the probability of change in one trait is dependent on the state of the other trait. (For example, the probability of a culture gaining shamanism can differ between cultures with a belief in an afterlife and cultures without one.) A likelihood ratio (LR) is used to compare the log likelihoods of the independent and dependent models. The advantage of this method is that its use is not conditioned on the ability to unequivocally reconstruct ancestral character states (Nunn 2011).

The supertree with the preferred set of divergence dates (i.e., shallower divergences) was used for correlation analysis. The probability that a model of dependent evolution fits the data significantly better than the model of independent evolution was estimated with a likelihood-ratio test involving 1000 Monte Carlo simulations. A likelihood-ratio test generates a null distribution of likelihood ratios, against which the significance of the observed LR is tested. For each simulation, maximum-likelihood estimates of model parameters were optimized using 500 iterations. If the dependent model fits the data significantly better than the independent model, this indicates that the state of one character affects the probability of change in the other, and that the two characters probably coevolve.

## Results

The resulting supertree topology (see ESM Figure A2) indicates a deep split between sub-Saharan African and non-African hunter-gatherers. Within Africa, South African Khoisan who speak !Kung and G/wi are more closely related to Central African Pygmies than to click-speaking East African Hadza and Sandawe. Outside Africa, the Vedda of Sri Lanka is the deepest-rooting lineage, followed by Andaman Islanders and Australian Aboriginals, presumably the remnants of early out-of-Africa expansion via the “southern route” (Macaulay et al. 2005). A large clade follows, consisting of two groups: hunter-gatherers of East Asia and those in Beringia and America. Within East Asia, two sister groups appear: Southeastern (Negritos of Malaysia and Philippines and Badjau Tawi) and Northeastern (the “Paleo-Asiatic” peoples). The Beringian-American clade consists of related Eskimo-Aleut and Na-Dene speakers, and the Amerind speakers of North and South America as a sister group of the two.

**Fig. 3:**
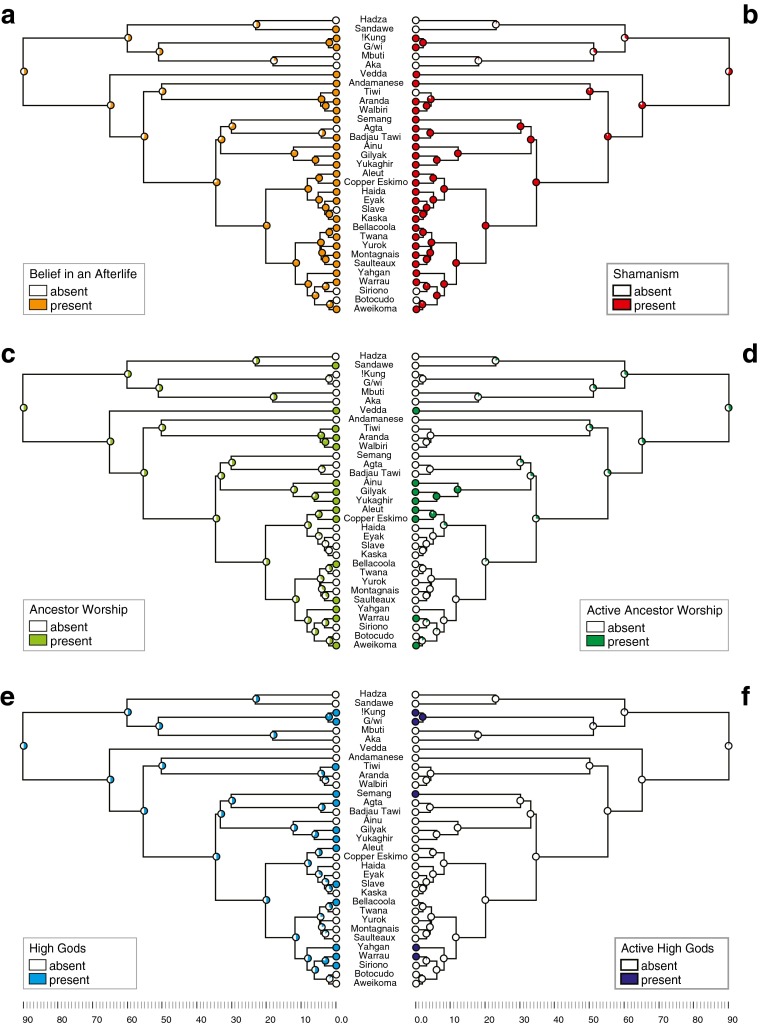
Maximum likelihood reconstructions of ancestral states for six characters describing hunter-gatherer religiosity. **a** Belief in an Afterlife. **b** Shamanism. **c** Ancestor Worship. **d** Active Ancestor Worship. **e** High Gods. **f** Active High Gods. The scale indicates time depth in kya. (see ESM Table A4 for details)

The reconstructions of ancestral states for religious beliefs and behaviors show several consistent patterns using maximum parsimony and maximum likelihood methods, and topologies with two alternative sets of divergence dates. The presence of animistic concepts in the religions of all sample societies (Fig. 2) is in accord with Tylor’s (1871) theory that animism is fundamental to religion. The presence of animism in the last common ancestor (LCA) of present-day hunter-gatherers is significantly supported (proportional likelihood = 0.99, *p* < 0.05*).^1^ Reconstructions of ancestral states for the six remaining characters describing religion in our hunter-gatherer sample are shown in Fig. 3. (Reconstructions of ancestral states for each node based on maximum parsimony and maximum likelihood using two sets of divergence dates are given in ESM Table A4.)

Belief in an afterlife and shamanism are present among 79% of sample societies (Fig. 2) and have similar, although not identical, distribution across societies (Fig. 3a, b). These characters are less common among African hunter-gatherers. The reconstructed ancestral state in the deepest node for belief in an afterlife and shamanism is equivocal (proportional likelihood = 0.5 and 0.56, respectively; ESM Table A4a) according to maximum likelihood. Maximum parsimony favors the absence of shamanism. We cannot determine whether belief in an afterlife and shamanic practices were present in the LCA of present-day hunter-gatherers. Among present-day African hunter-gatherers (the deepest-rooting clades) the !Kung and G/wi hold a belief in an afterlife, whereas Hadza, Mbuti, and Aka either never acquired it or had the trait and then lost it (Fig. 3a). The !Kung and G/wi have shamans, but Hadza, Sandawe, Aka, and Mbuti do not (Fig. 3b). The presence of healers among the Mbuti suggests that the Mbuti may once have had shamans, but they lost the trait at some point (Winkelman 1990).

Ancestor worship is present in 45% and active ancestor worship in 24% of sample societies (Fig. 2). The ancestral state of “ancestor worship” is equivocal (proportional likelihood = 0.5; ESM Table A4b). Ancestral presence of active ancestor worship is somewhat less likely. However, this is not significantly supported (proportional likelihood = 0.43429948; ESM Table A4b) on the topology with shallower divergence dates. In contrast, its ancestral absence is significantly supported (0.10739427*; ESM Table A4b) on the topology with deeper divergence dates. Maximum parsimony reconstruction favors the absence of active ancestor worship (ESM Table A4b). These results suggest ancestor worship could have been present among ancestral hunter-gatherers, but probably not the active form.

Only 39% of sample societies have the trait “high gods,” and even fewer (15%) have active high gods (Fig. 2). The equivocal results based on maximum likelihood and absence according to maximum parsimony for the ancestral state of “high gods” suggests possible presence of belief in a single creator deity among ancestral hunter-gatherers, albeit one that is not active in human affairs (proportional likelihood = 0.05*) (see ESM Table A4b for details).

The consistency and retention indices (CI, RI) calculated for each character and the whole character matrix quantifies the degree of character “fit” on the tree. CI (with values from 0 to 1) measures the amount of homoplasy on a tree; RI (also 0–1) measures the degree to which shared derived character states are exhibited on a tree. The resulting CI and RI values for the whole character matrix are low (0.17 and 0.31, respectively), indicating that most characters are highly labile. The characters displaying the worst fit on phylogeny are ancestor worship and high gods (CI = 0.1), and the reconstructed ancestral states for both are equivocal (Fig. 3c, e; ESM Table A4b).

**Fig. 4:**
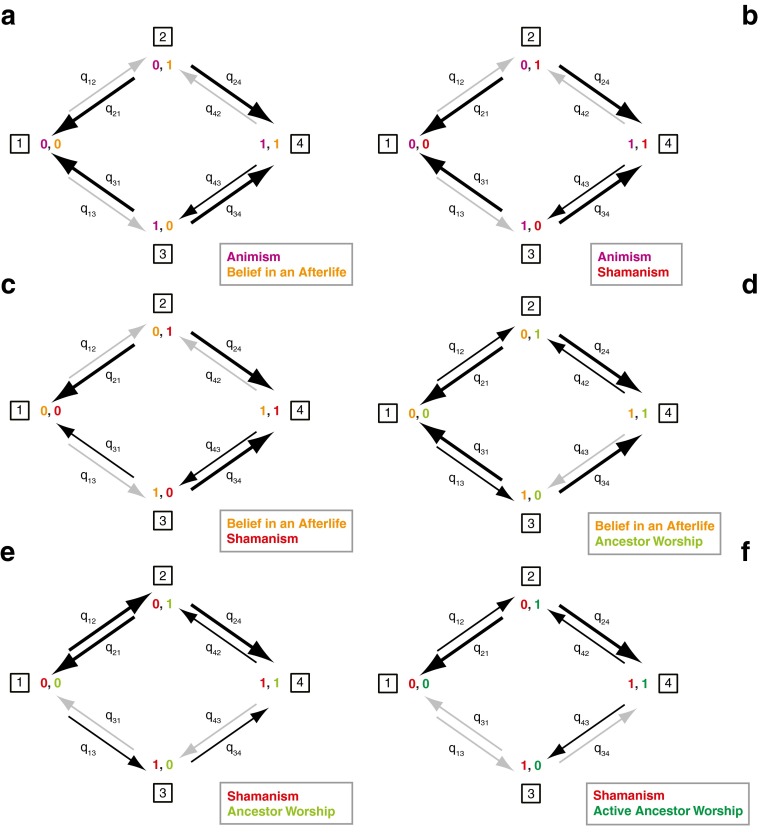
Transitions between character states for selected pairs of characters showing significantly higher likelihood of the dependent model of evolution, indicating that these traits coevolve. Widths of arrows are proportional to rates of change (see ESM Table A5 for details)

The results of Pagel’s test for correlated evolution indicate a significant positive relationship between most traits investigated. The dependent models for the evolution of selected pairs of characters showing support for correlated evolution are shown in Fig. 4. (The results of Pagel’s test for correlated evolution, including transition rates for independent and dependent model of character change, log likelihood values for each model, and *p* values, are given in ESM Table A5.)

Belief in an afterlife and shamanism emerge in the presence of the fundamental trait of animism. Once these two traits are gained, they are unlikely to be lost (Fig. 4a).

Coevolution of belief in an afterlife and shamanism is significantly supported (L_i_ = −35.71, L_d_ = −28.75, LR = 6.96, *p* = 0.00*) (Fig. 4c). The transitional probabilities indicate that belief in an afterlife evolves more likely in the absence of shamanism than shamanism would evolve in the absence of a belief in an afterlife. This suggests that belief in an afterlife is likely to have emerged first from the base of animistic beliefs, and later shamanism evolved in the presence of belief in an afterlife (Fig. 4c). It also indicates that shamanism is likely to be lost in the absence of belief in an afterlife (Fig. 4c; ESM Table A5).

There is significant support for coevolution of belief in an afterlife and ancestor worship (L_i_ = −40.48, L_d_ = 34.66, LR = 5.82, *p* = 0.003*) (Fig. 4d). Belief in an afterlife evolves prior to ancestor worship, and its presence stimulates the subsequent evolution of ancestor worship. Ancestor worship is unlikely to be lost in the presence of belief in an afterlife (Fig. 4d).

There is also significant support for coevolution of both shamanism and ancestor worship (L_i_ = −40.48, L_d_ = −36.60, LR = 3.88, *p* = 0.01*) and shamanism and active ancestor worship (L_i_ = −34.74, L_d_ = −28.72, LR = 6.02, 0.001*). Shamanism seems to have a deep history and continuity, whereas ancestor worship, although it could have evolved very early in the history of modern humans, is a highly labile trait (Fig. 3c). Active ancestor worship probably appeared later (Fig. 3d). Ancestor worship without shamanism seems to be an unstable cultural state that results either in a loss of worshipful relationship with dead kin or in the appearance of the shaman. Ancestor worship with shamanism, on the other hand, appears to be a stable cultural state, rarely lost once achieved, and the same is seen for active ancestor worship with shamanism (Fig. 4e, f).

There is no support for coevolution of any pair of characters that includes high gods and active high gods, with the obvious exception of the two traits themselves (L_i_ = −33.58, L_d_ = −27.93, LR = 5.65, *p* = 0.001). Surprisingly, not even belief in an afterlife shows any correlation with high gods. Belief in an afterlife evolved prior to high gods, as is evident from reconstruction of ancestral states and the transition rates based on Pagel’s test for correlated evolution. But these pairs of characters do not coevolve: in other words, the probability of change in one is not affected by the state of the other (see ESM Table A5 for details).

## Discussion

Our results reflect Tylor’s (1871) belief that animism was the earliest and most basic trait of religion because it enables humans to think in terms of supernatural beings or spirits. Animism is not a religion or philosophy, but a feature of human mentality, a by-product of cognitive processes that enable social intelligence, among other capabilities. It is a widespread way of thinking among hunter-gatherers (Bird-David 1999; Charlton 2007; Klingensmith 1953; Piaget 1929). Animistic thought is a natural by-product of the human capacity for intentionality or “theory of mind mechanism” (Dunbar 2003). This innate cognitive trait allows us to attribute a vital force to animate and inanimate elements in the environment (Piaget 1929; Tylor 1871). Once that vital force is assumed, attribution of other human characteristics will follow. Animistic beliefs are generally adaptive in the environments that prevail in hunter-gatherer societies (Bird-David 1999; Charlton 2002). Animistic thinking would have been present in early hominins, certainly earlier than language (Coward 2015; Dunbar 2003).

It can be inferred from the analyses, or indeed from the universality of animism, that the presence of animistic belief predates the emergence of belief in an afterlife. Once animistic thought is prevalent in a society, interest in the whereabouts of spirits of the dead could reasonably lead to the concept of an unseen realm where the individual personality of the deceased lives on. The afterlife might be a rewarding continuation of life on earth, or a realm of eternal punishment for those who break social norms. Belief in an afterlife may have generated a sense of “being watched” by the spirits of the dead, prompting archaic forms of social norms (Bering 2006) actualized in the role of the shaman.

Shamanism significantly correlates with belief in an afterlife, which emerged first. Shamanism then evolved in the presence of belief in a realm of spirits of the dead. If belief in an afterlife is lost, shamanism is also likely to be lost. The single exception to this in our sample is the Slave, who have shamanism without belief in an afterlife. Although shamanism has been described as the universal religion of Paleolithic hunter-gatherers (Eliade 1964; Winkelman 1990), it is not a religion per se, but a complex of beliefs and behaviors that focus on communication with the ancestral spirits, as well as the general world of spirits in the realm of the afterlife. Shamans are healers, ritual leaders, and influential members of society whose keen insight and success in solving social problems (Rossano 2007; Winkelman 1990) can lead to wealth, power, and access to mates. Shamanism acts as a mechanism to reinforce social norms, encouraging group cooperation through ritual and social bonding, and calming anxiety during times of resource stress (Hayden 1987; Rossano 2007; Winkelman 1990; Winkelman 2010). Shamans, as Vitebsky (2000) puts it, are both spiritual leaders and social workers. It would be reasonable to argue that shamans, who draw their power from communication with the world of spirits, would have initially emerged in strongly animistic societies that believed in an afterlife. Communication with omniscient and perhaps judgmental spirits of known deceased, including ancestors, would have been a useful tool in the work of the shaman.

As humans migrated out of Africa more than 60 kya (Henn et al. 2012; Macaulay et al. 2005), the shaman’s curing skills and group rituals would have enhanced survival through physical and emotional healing, enforcement of group norms, and resource management. At the time of the rapid population dispersal of AMH out of Africa along the southern route into Wallacea and Sahul, the physical stress of travel and encounters with unfamiliar cultures in areas already occupied by other hominins would have driven the need for use of both material and non-material culture, including religion, to negotiate identities and relationships among and between groups (Coward 2015). Evidence for more complex information exchange systems, planning depth and authority, and increased symbolization appears in the archaeological record of Wallacea and Australia prior to 40 kya (Balme et al. 2009). These suggestions are in line with the elevated likelihood support of the ancestral presence of shamanism in the deepest out-of-Africa nodes (Fig. 2b). Our results support Rossano’s (2009a) hypothesis that shamanism preceded and is more basic than ancestor worship, although the presence of shamanism in the LCA of present-day hunter-gatherers is not supported.

Despite established speculation by Spencer (1870) and Tylor (1871) that universal ancestor worship was the rudimentary beginning of religion, our analysis shows that worship of dead kin is neither widespread among hunter-gatherers nor the oldest trait of religion. Fewer than half of the societies in our sample believe that dead kin can influence the living (Fig. 2). In many hunter-gatherer societies the concept of ancestor spirits is absent, or present but they are inactive in human affairs (Sheils 1975; Swanson 1960). For example, among the! Kung there is a general fear of active spirits of “the dead,” who are often the ghosts of recently deceased kin. But the concept of having a worshipful relationship with their own ancestors is absent (Marshall 1962). Greater likelihood of the presence of active ancestor worship has been linked to societies with unilineal descent where important decisions are made by the kin group (Sheils 1975; Swanson 1960). Ancestor worship is an important source of social control that strengthens cohesion among kin and maintains lineal control of power and property (Sheils 1975; Steadman et al. 1996; Swanson 1960), particularly in the more complex hunter-gatherer societies. In contrast, immediate-return hunter-gatherer societies (Woodburn 1982) seldom recognize dead ancestors who may intervene in their lives. The social structure of these societies does not usually consist of strong kin ties, and individuals do not depend on help from close kin, living or dead (Barnard and Woodburn 1988).

The minimum requirement for veneration of dead ancestors is animism and belief in the survival of the personal identity beyond death. In our analyses, ancestor worship is significantly positively related with belief in an afterlife and shamanism. Belief in an afterlife evolves prior to shamanism and ancestor worship. There is significant support for coevolution of shamanism with ancestor worship and active ancestor worship. Belief in an afterlife with shamanism appears to be a stable cultural state, rarely lost once achieved. Ancestor worship is also less likely to be lost in the presence of belief in an afterlife with shamanism.

This is not to say that the reduction of complexity of religious beliefs and behaviors cannot occur in simple hunter-gatherers. The presence of belief in an afterlife and shamanism is significantly supported in the LCA of Beringian-American as well as North and South American hunter-gatherers (Fig. 3a,b; ESM Table A4), suggesting that the absence of these traits among the Siriono in Bolivia and Botocudo in southern Brazil is due to independent secondary losses. In the Siriono, this loss was probably part of a substantial decrease in cultural complexity during the expansion of Tupi language speakers across lowland South America (Walker et al. 2012).

It can be argued that those societies under higher resource stress, encountering difficulties with resource extraction that demands cooperative effort, would benefit most from the shaman’s skills (Hayden 1987; Rossano 2007; Winkelman 1990, 2010). This is supported by the prevalence of shamanism among hunter-gatherer societies of Eurasia, and corresponding support for the presence of shamanism among their ancestors (Fig. 2b). In “Paleo-Asiatic” peoples (Ainu, Gilyak, and Yukaghir) and in Eskimo-Aleut peoples of the circumpolar region, the presence of shamanism combines with active ancestor worship. The presence of both traits in the LCAs of these groups is significantly supported (Fig. 2d). High gods were not the first supernatural entities to monitor morality (Geertz 2014). The power and leadership of the shaman was often based on reaffirming traditional social behavior that was presumed to have been carried out by the ancestors, and still desired and monitored by punishing ancestral spirits, even in those societies where spirits of dead kin were not considered a part of the religion (Steadman and Palmer 2008).

Belief in high gods appears to be a rather “stand-alone” phenomenon in the evolution of hunter-gatherer religion. Prior studies have shown that among the four modes of subsistence (hunter-gatherers, pastoralists, horticulturalists, and agriculturalists) hunter-gatherers are least likely to adopt morally punishing active high gods, if any high gods at all (Botero et al. 2014; Norenzayan 2013; Peoples and Marlowe 2012; Swanson 1960). This pattern is reflected in the distribution and the reconstructed evolution of high gods and active high gods in our sample (Fig. 2; Fig. 3e, f). Early egalitarian hunter-gatherers would rarely have acknowledged an active high god (Norenzayan 2013; Peoples and Marlowe 2012) and would be the least likely to accept or benefit from the supernatural meddling and social constraints of deities who would be seen as “high rulers” (Peoples and Marlowe 2012). The leaders of complex hunter-gatherer societies whose subsistence relies on collective effort should be more likely to benefit from the coercive power of a punishing high god. Our analysis does not support the prevalence of either type of high god among ancestral hunter-gatherers, and the evolution of high gods does not correlate with any of the other traits of hunter-gatherer religion, including ancestor worship.

In a study by Guglielmino et al. (1995) analyzing the transmission pattern of cultural traits in sub-Saharan Africa, high gods (Swanson 1960) was among a group of traits (taboos, ritual mutilation, premarital norms, etc.) that were consistently the least correlated with either language or ecology, suggesting they evolve rapidly and are prone to cultural borrowing. On the other hand, according to a more recent study by Currie and Mace (2014), high gods are among those cultural traits that evolve at relatively slow rates in Bantu and Austronesian societies. Our results are consistent with Guglielmino et al. (1995) since high gods (and ancestor worship) show the worst fit on phylogeny (CI = 0.1) of all studied characters. This suggests that the presence of high gods and some other traits related to religion and ritual are influenced by more socioculturally oriented factors, and it lends support to the idea that these types of traits may be more labile. Such traits would be more readily gained or lost as the adaptively relevant sociopolitical environment changes (Irons 1998). To some extent this finding may explain the independent pattern of emergence of high gods in our study.

Ancestral spirits and local gods with limited powers of supernatural monitoring may have come relatively easily to the minds of early human hunter-gatherers. These types of supernatural entities operate in a different realm from omniscient and powerful creator gods (high gods), who have been shown to be related to a culture of some type of control or decision-making structure (Peoples and Marlowe 2012; Radin 1937; Swanson 1960). The absence of belief in active gods and spirits in the LCA of present-day hunter-gatherers, according to the reconstructions, indicates a deep evolutionary past for the egalitarian ethos of most simple hunter-gatherer societies, whose small mobile populations of self-sufficient individuals make collective action problems less of an issue. Those societies would be the least likely to accept or benefit from the personal restraints of active ancestors or active high gods.

## Conclusion

In this study we used a suite of phylogenetic comparative methods to investigate the early evolution of religion. We reconstructed ancestral states for seven characters describing religious beliefs and behaviors in a global sample of 33 hunter-gatherer societies and tested for correlated evolution between these characters and for the direction of cultural change.

Our results indicate that the oldest trait of religion, shared by the most recent common ancestor of present-day hunter-gatherers, was animism. This supports long-standing beliefs about the antiquity and fundamental role of this component of human mentality, which enables people to attribute intent and lifelike qualities to inanimate objects and would have prompted belief in beings or forces in an unseen realm of spirits**.** Reconstructions are equivocal on whether or not the religion of the LCA of present-day hunter-gatherers included belief in an afterlife, shamanism, ancestor worship, and the concept of a single creator deity, or a high god. Belief in either ancestral spirits or creator deities who remain active in human affairs was not present in ancestral hunter-gatherer societies, according to the reconstructions. This may be indicative of a deep past for the egalitarian nature of hunter-gatherer societies, to whom high gods would appear to be rulers (Peoples and Marlowe 2012).

The majority of traits of religion we investigated exhibit a correlated pattern of character change on phylogeny. The results suggest that belief in an afterlife, shamanism, and ancestor worship evolve in concerted fashion as an integrated system of beliefs and practices. However, neither high gods nor active high gods exhibit correlated evolution with the rest of the religious traits, including ancestor worship, despite Spencer’s and Tylor’s suggestions.

This is in line with a variety of evidence from other studies (Botero et al. 2014; Norenzayan 2013; Peoples and Marlowe 2012; Radin 1937; Swanson 1960) suggesting that if a society acquires belief in an omniscient and potentially morally punishing creator deity, it does so regardless of other aspects of its religion but more as a reflection of its social and political structure.

## Electronic supplementary material

ESM 1 (PDF 329 kb)

## Footnotes

1 Asterisk (*) indicates significant result *p* ≤ 0.05
