# Supplementary material for: Hunter-Gatherers and the Origins of Religion
Source: Hum Nat. 2016 May 6;27:261–82. doi: 10.1007/s12110-016-9260-0 (PMC4958132; doi:10.1007/s12110-016-9260-0)
Supplement: Supplementary file 1 — (PDF 329 kb) [file 12110_2016_9260_MOESM1_ESM.pdf]

Hunter-Gatherers and the Origins of Religion

Hervey C. Peoples, Pavel Duda, and Frank W. Marlowe

Electronic Supplementary Materials (ESM)

Human Nature 27(3), doi: 10.1007/s12110-016-9260-0

Contents

Methods 2

References........ 3

Table A1a. Sample of hunter-gatherer societies: matrix of religiosity characters 4

Table A1b. References for 33 hunter-gatherer societies religiosity characters 5

Table A2. Hunter-gatherer populations in the study sample, their taxonomic nomenclature, representation across source trees, and proxies selected for hunter-gatherer populations absent in source trees. 8

Figure A1. Linguistic scaffold for supertree typology 10

Figure A2. Supertree topology used in the study 10

Table A3a. Divergence table of hunter-gatherer populations in the study sample........ 11

Table A3b. References for divergence dates for hunter-gatherer populations in the study sample 17

Table A4a. Ancestral reconstruction for three characters of hunter-gatherer religiosity in all nodes: Animism, Afterlife, Shamanism........ 21

Table A4b. Ancestral reconstruction for four characters of hunter-gatherer religiosity in all nodes: Ancestor Worship, High Gods, Active High Gods and Ancestor Worship 25

Table A5. Pagel's test for correlated discrete character evolution 29

Methods

Supertree dataset manipulations

Some sample populations are underrepresented or absent from the supertree dataset. Many hunter-gatherer populations live in areas that are difficult to access and some of them, e.g., Vedda, Botocudo, and Yahgan, are nowadays extinct or near-extinct. Taxa possessing insufficient amount of character information can act as so called 'wildcard' taxa *sensu* (Nixon and Wheeler 1993). These taxa adopt multiple positions in optimal topologies leading to poorly resolved consensus trees with large polytomies that hamper the interpretation and further utilization of the phylogenetic results. Population absent from the supertree dataset were replaced by more inclusive (i.e., higher level) taxon or by genetically closely related taxon (population) present in the dataset that was used as a proxy for the population in question (ESM Table A2). Positions of four North American populations – Kaska, Eyak, Twana, and Yurok – were based solely on linguistic classification (ESM Table A2). Six populations not present in the source trees for which sufficient linguistic classification was not available – Ingalik, Micmac, E. Pomo, Yokuts (Lake), Klamath, and Kutenai – were excluded from the dataset. The analysis of most parsimonious trees (MPTs) using the IterPCR script (Pol and Escapa 2009) implemented in TNT (Goloboff et al. 2008) identified these populations as wildcard taxa, supporting their exclusion.

Some hunter-gatherer populations act as wildcard taxa because of conflicting information implied by the source trees. This conflict is often caused by recent genetic admixture with (often distantly related) immigrant populations. For example Aleut of southwestern Alaska who have undergone a pervasive admixture with Russian colonizers and Scandinavian and English fishermen since the Russian contact in 1741. As a consequence, underlying patterns of genetic structure of the Aleut population are obscured (Rubicz et al. 2010a, b). Genetic admixture between hunter-gatherers and agriculturalists is often sex-biased as documented for Central African Pygmies and Bantu immigrants (Batini et al. 2011; Quintana-Murci et al. 2008; Verdu et al. 2009), causing conflicts between source trees based on maternally, paternally, and biparentally inherited genetic markers. Contacts between hunter-gatherers and agriculturalists are often followed by some degree of cultural assimilation that can include language shifts (Bahuchet 2012).

In order to overcome the problem of the lack of data and the conflicting signals caused by recent genetic admixture and language shifts in some hunter-gatherer populations in the study sample, the characters based on linguistic classifications were up-weighted by a factor of 100 to serve as a topological constraint or 'scaffold'. This 'linguistic scaffold' (compare to 'molecular scaffold' *sensu* Springer et al. (2001)) constrains the topology for a subset of populations for which linguistic affiliation can be determined (i.e., those scored for characters). Language isolates according to Ethnologue classification (Lewis et al. 2013) were scored entirely using '?'. Languages of American hunter-gatherers classified were classified as Amerindian (Ruhlen 1991) merely to ensure their presence within the American clade.

Hunter-gatherer populations speaking languages of agriculturalists as a result of relatively recent language shift were scored entirely using '?' in the scaffold tree. This allowed these populations to adopt a position on the MRP supertree based on contributing source trees alone. These populations include Mbuti and Aka Pygmies who speak Niger- Kordofanian and Nilo-Saharan languages (Bahuchet 2012), Vedda of Sri Lanka who speak Indo-European language (Dharmadasa 1974), Semang, the Negritos of Malaysia who speak Austro-Asiatic (Aslian) language (Burenhult et al. 2011; Dunn et al. 2013), and Agta, the Negritos of Philippines who speak Malayo-Polynesian (Austronesian) language (Reid 2013).

The linguistic scaffold tree included 20 phylogenetically informative characters for the 33 populations in the study sample. Note that this linguistic scaffold implied relatively few internal groupings (clades based on linguistic classification), particularly among the Old World hunter-gatherers (ESM Fig. A1).

References

Bahuchet, S. (2012). Changing Language, Remaining Pygmy. *Human Biology, 84*(1), 11–43.

Batini, C., Lopes, J., Behar, D. M., Calafell, F., Jorde, L. B., van der Veen, L., et al. (2011). Insights into the Demographic History of African Pygmies from Complete Mitochondrial Genomes. *Molecular Biology and Evolution*, *28*(2), 1099–1110.

Burenhult, N., Kruspe, N., & Dunn, M. (2011). Language history and culture groups among Austroasiatic-speaking foragers of the Malay Peninsula. In N. J. Enfield (Ed.), *Dynamics of Human Diversity: The Case of Mainland Southeast Asia* (pp. 257–275). Canberra: Pacific Linguistics.

Dharmadasa, K. (1974). The creolization of an aboriginal language: The case of Vedda in Sri Lanka (Ceylon). *Anthropological Linguistics*, *16*(2), 79–106.

Dunn, M., Kruspe, N., & Burenhult, N. (2013). Time and Place in the Prehistory of the Aslian Languages. *Human Biology, 85*(1–3), 383–399.

Goloboff, P. A., Farris, J. S., & Nixon, K. C. (2008). TNT, a free program for phylogenetic analysis. *Cladistics*, *24*(5), 774–786.

Greenberg, J. H., & Ruhlen, M. (2007). *An Amerind Etymological Dictionary*. Stanford, CA: Stanford University Press.

Lewis, M., Simons, G., & Fennig, C. (2013). *Ethnologue: Languages of the world.* (Seventeenth ed.). Dallas, TX: SIL International. Available online at *http://www.ethnologue.com*

Nixon, K. C., & Wheeler, Q. D. (1993). Extinction and the origin of species. In M. J. Novacek, & Q. D. Wheeler (Eds.), *Extinction and Phylogeny* (pp. 119–143). New York: Columbia University Press.

Pol, D., & Escapa, I. H. (2009). Unstable taxa in cladistic analysis: identification and the assessment of relevant characters. *Cladistics, 25*(5), 515–527.

Quintana-Murci, L., Quach, H., Harmant, C., Luca, F., Massonnet, B., et al. (2008). Maternal traces of deep common ancestry and asymmetric gene flow between Pygmy hunter-gatherers and Bantu-speaking farmers. *Proceedings of the National Academy of Sciences, 105*(5), 1596–1601.

Reid, L. A. (2013). Who Are the Philippine Negritos? Evidence from Language. *Human Biology,* 85(1–3), 329–358.

Rubicz, R., Melton, P. E., Spitsyn, V., Sun, G. Y., Deka, R., & Crawford, M. H. (2010a). Genetic Structure of Native Circumpolar Populations Based on Autosomal, Mitochondrial, and Y Chromosome DNA Markers. *American Journal of Physical Anthropology*, *143*(1), 62–74.

Rubicz, R., Zlojutro, M., Sun, G., Spitsyn, V., Deka, R., Young, K. L., et al. (2010b). Genetic Architecture of a Small, Recently Aggregated Aleut Population: Bering Island, Russia. *Human Biology*, *82*(5–6), 719–736.

Ruhlen, M. (1991). *A guide to the world's languages: classification* (Vol. 1). Stanford, CA: Stanford University Press.

Springer, M. S., Teeling, E. C., Madsen, O., Stanhope, M. J., & de Jong, W. W. (2001). Integrated fossil and molecular data reconstruct bat echolocation. *Proceedings of the National Academy of Sciences of the United States of America, 98*(11), 6241–6246.

Verdu, P., Austerlitz, F., Estoup, A., Vitalis, R., Georges, M., Théry, S., et al. (2009). Origins and Genetic Diversity of Pygmy Hunter-Gatherers from Western Central Africa. *Current Biology, 19*(4), 312–318.

Table A1a. Sample of hunter-gatherer societies: matrix of religiosity characters

SCCS/ **EA** Number	Society Name	References	Animism	Afterlife	Shamanism	Ancestor Worship	High Gods	Active Ancestor Worship	Active High Gods
2	!Kung	1–7	1	1	1	0	1	0	1
—	G/wi	8, 9	1	1	1	0	1	0	1
9	Hadza	1, 10	1	0	0	0	0	0	0
**301**	Sandawe	11	1	1	0	1	0	0	0
13	Mbuti	1–3, 12, 13	1	0	0	0	0	0	0
—	Aka	14, 15	1	0	0	0	0	0	0
77	Semang	1–4,16, 17	1	1	1	0	1	0	1
79	Andamanese	1, 4, 18	1	1	1	0	0	0	0
80	Vedda	1, 4, 19, 20	1	1	1	1	0	1	0
86	Badjau Tawi	21, 22	1	1	1	0	0	0	0
—	Agta	23, 24	1	0	1	0	1	0	0
90	Tiwi	1, 25–27	1	1	0	1	1	0	0
91	Aranda	1, 4, 28–30	1	1	1	1	0	0	0
**1177**	Walbiri	31	1	1	1	1	0	0	0
118	Ainu	1, 32–35	1	1	1	1	0	1	0
119	Gilyak	1, 36, 37	1	1	1	1	1	1	0
120	Yukaghir	38	1	1	1	1	1	1	0
123	Aleut	1, 39–41	1	1	1	1	1	1	0
124	Copper Eskimo	1, 28, 42–46	1	1	1	1	0	1	0
125	Montagnais	1–3, 47–49	1	1	1	0	0	0	0
127	Salteaux	1, 4, 50, 51	1	1	1	1	0	0	0
128	Slave	1, 52	1	0	1	0	1	0	0
129	Kaska	1–3, 53, 54	1	1	1	0	0	0	0
130	Eyak	1, 55	1	1	1	0	0	0	0
131	Haida	1, 56–61	1	1	1	0	0	0	0
132	Bellacoola	1, 62, 63	1	1	1	1	1	0	0
133	Twana	1–3, 64, 65	1	1	1	0	0	0	0
134	Yurok	1, 28, 64, 66	1	1	1	0	0	0	0
162	Warrau	1, 67, 68	1	1	1	1	1	1	1
173	Siriono	1–3, 69	1	0	0	0	1	0	0
178	Botocudo	4, 70, 71	1	0	0	0	0	0	0
180	Aweikoma	1, 4, 72–74	1	1	1	1	0	1	0
186	Yahgan	1, 28, 75	1	1	1	1	1	0	1

Present = 1, absent = 0

Table A1b. References for 33 hunter-gatherer societies religiosity characters

1. Murdock G. P., & White, D. R. (1980). Standard cross-cultural sample. In H. Barry & A. Schlegel (Eds.), *Cross-cultural samples and codes* (pp. 3–43). Pittsburgh: University of Pittsburgh Press.
2. Winkelman, M. (1990). Shamans and other "magico-religious" healers: a cross-cultural study of their origins, nature, and social transformations. *Ethos*, *18*(3), 308–352.
3. Winkelman, M. & White, D. (1987). A cross-cultural study of magico-religious practitioners and trance states: data base. In D. Levinson and R. Wagner (Eds.), *Human relations area files research series in quantitative cross-cultural data* (Vol. 3). New Haven: HRAF Press.
4. Sheils, D. (1975). Toward a unified theory of ancestor worship: a cross-cultural study. *Social Forces, 54*(2), 427–440.
5. Marshall, L. (1962). !Kung bushman religious beliefs. *Africa: Journal of the International African Institute, 32*(3), 221–252.
6. Marshall, L. (1969). The medicine dance of the !Kung Bushmen. *Africa: Journal of the International African Institute*, *39*(4), 347–381.
7. Marshall, L. (1976*). The !Kung of Nyae Nyae*. Cambridge, MA: Harvard University Press.
8. Barnard, A. (1992). *Hunters and herders of Southern Africa: a comparative ethnography of the Khoisan peoples.* Cambridge, UK: Cambridge University Press.
9. Silberbauer, G. B. (1981). *Hunter and habitat in the central Kalahari Desert*. Cambridge: Cambridge University Press.
10. Marlowe, F. W. (2010). *The Hadza: hunter-gatherers of Tanzania*. Berkeley: University of California Press.
11. Raa, E. T. (1969). The moon as a symbol of life and fertility in Sandawe thought. *Africa*: *Journal of the International African Institute, 39*(1), 24–53.
12. Turnbull, C. M. (1965). The Mbuti Pygmies: an ethnographic survey. *Anthropological Papers of the AMNH*, *50*, 139-282.
13. Turnbull, C. M. (1965). *Wayward servants: the two worlds of the African Pygmies*. Garden City, NY: The Natural History Press.
14. Hewlett, B. S. (1993*). Intimate fathers: the nature and context of Aka Pygmy paternal infant care*. Ann Arbor: University of Michigan Press.
15. Sawada, M. (2001). Rethinking methods and concepts of anthropological studies on African Pygmies' world view: the creator-god and the dead. *African Study Monographs, 27* (supplementary issue), 29–42.
16. Endicott, K. M. (1979). *Batek Negrito religion: the world-view and rituals of a hunting and gathering people of Peninsular Malaysia*. Oxford: Oxford University Press.
17. Schebesta, P., & Schutze, F. (1957). *The Negritos of Asia*. Wien-Mödling: St.-Gabriel- Verlag.
18. Radcliffe-Brown, A. R. (1922). *The Andaman Islanders: a study in social anthropology*. Cambridge: Cambridge University Press.
19. Bailey, J. (1863). An account of the wild tribes of the Veddahs of Ceylon: their habits, customs, and superstitions. *Transactions Ethnological Society of London*, *2*, 278–320.
20. Seligman, C. G., & Seligman, B. Z. (1911). *The Veddas*. Cambridge: Cambridge University Press.
21. Nimmo, H. A. (1972). *Badjau of the Philipines*. New Haven: HRAF Press.
22. Nimmo, H. A. (1965). Social organization of the Tawi-Tawi Badjaw. *Ethnology, 4*(4), 421–439.
23. Headland, T. N. (1987). Kinship and social behavior among Agta Negrito hunter- gatherers. *Ethnology*, *6*(4), 261–280.
24. Rahmann, R., & Maceda, M. N. (1955). Notes on the Negritos of Northern Negros. *Anthropos, 50*(4/6), 810–836.
25. Goodale, J. C. (1971). *Tiwi wives: a study of the women of Melville Island, North Australia.* Seattle: University of Washington Press.

26. Goodale, J. C. (1999). The Tiwi of Melville and Bathurst Islands, north Australia. In R. B. Lee, & R. H. Daly (Eds.), *Cambridge encyclopedia of hunters and gatherers* (pp. 353–362). Cambridge, UK: Cambridge University Press.
27. Hart, C. W. M. (1930). *The Tiwi of Melville and Bathurst Islands*. Sydney: University of Sydney.
28. Swanson, G .E. (1960). *The birth of the gods: the origin of primitive belief*. Ann Arbor: University of Michigan Press.
29. Spencer, S. B., & Gillen, F. J. (1938 [1899]). *The native tribes of Central Australia*. London: Macmillan.
30. Penniman, T. K. (1929). The Arunta religion. *The Sociological Review*, 21, 10–37.
31. Meggitt, M. J. (1965). *Desert people: a study of the Walbiri Aborigines of Central Australia*. Chicago: University of Chicago Press.
32. Watanabe, H. (1964). *The Ainu: a study of ecology and the system of social solidarity between man and nature in relation to group structure*. Tokyo: University of Tokyo.
33. Batchelor, J. (1927. *Ainu life and lore: Echoes of a departing race*. Tokyo: Kyobunkwan.
34. Ohnuki-Tierney, E. (1973). The shamanism of the Ainu of the northwest coast of Southern Sakhalin. *Ethnology*, *12*(1), 15–29.
35. Munro, N. G., Seligman, B. Z., & Watanabe, H. (1963). *Ainu creed and cult*. New York: Columbia University Press.
36. Black, L. (1973). The Nivkh (Gilyak) of Sakhalin and the Lower Amur. *Arctic Anthropology, 10*(1), *1–112.*
37. Seeland, N., & Schütze, F. (1882). The Gilyaks: an ethnographic sketch. *Russische Revue*, *21*, 97-130, 222–254.
38. Jochelson, W. (1926). The Yukaghir and the Yukaghirized Tungus. In F. Boas (Ed.), *The Jesup North Pacific expedition* (Vol. 9, pp. 135–342). Leiden, NL: Brill.
39. Sarychev, G. A. (1806*). Account of a voyage of discovery to the north-east of Siberia, the Frozen Ocean, and the North-East Sea* (Vol. 2). London: Printed for R. Phillips by J. G. Barnard.
40. Lantis, M. (1984). Aleut. In D. Damas (Ed.), *Arctic* (pp. 161–184). Washington, D.C: Smithsonian Institution.
41. Jones, D. M. (1970. *A study of social and economic problems in Unalaska, an Aleut village*. Ann Arbor: University Microfilms. .
42. Jenness, D. (1922). *The life of the Copper Eskimos*. Ottawa: F.A. Acland.
43. Jenness, D. (1959). *The people of the twilight*. Chicago: University of Chicago Press.
44. Damas, D. (1972). The Copper Eskimo. In M. G. Bicchieri (Ed.), *Hunters and gatherers today: a socioeconomic study of eleven such cultures in the Twentieth Century* (pp. 3– 50). New York: Holt, Rinehart and Winston..
45. De Coccola, R., King, P., & Houston, J. (1986). *The incredible Eskimo: life among the barren land Eskimo*. Surrey, B.C.: Hancock House.
46. Jenness, D. (1917). The Copper Eskimos. *Geographical Review, 4*, 81–91.
47. Desbarats, P. (1969). *What they used to tell about: Indian legends from Labrador*. Toronto: McClelland and Stewart Limited.
48. Lane, K. S. (1952). The Montagnais Indians, 1600–1640. *Kroeber Anthropological Society papers, 7*, 1-62.
49. Speck, F. G. (1935). *Naskapi: the savage hunters of the Labrador Peninsula*. Norman: University of Oklahoma Press.
50. Hallowell, A. I. (2002). Ojibwa ontology, behavior, and world view. In. G. Harvey (Ed.), *Readings in indigenous religion* (pp. 17–49). London: Continuum.
51. Dunning, R. W. (1959). *Social and economic change among the northern Ojibwa*. Toronto: University of Toronto Press.
52. MacNeish, J. H. (1954). Folk beliefs of a Slave Indian band. *Journal of American Folklore,*

67(264), 185–198.
53. MacNeish, J. H., & Teit, J. A. (1956). Field notes on the Tahltan and Kaska Indians: 1912–1915. *Anthropologica*, *3*(1), 40–171.
54. Honigmann, J. J. (1949). *The Kaska Indians: culture and ethos of Kaska society*. New Haven: Yale University Press.
55. Birket-Smith, K., & De Laguna, F. (1938). *The Eyak Indians of the Copper River Delta, Alaska.* Copenhagen: Levin & Munksgaard.
56. Tylor, E. B. (1899). On two British Columbian house-posts with totemic carvings, in the Pitt-Rivers Museum, Oxford. *The Journal of the Anthropological Institute of Great Britain and Ireland, 28*(1/2), 136–137.
57. Stevenson, I. (1975). The belief and cases related to reincarnation among the Haida. *Journal of Anthropological Research*, *31*(4), 364–375.
58. Murdock, G. P. (1934). Kinship and social behavior among the Haida. *American Anthropologist, 36*(3), 355–385.
59. Kan, S. (1986). The 19th-Century Tlingit potlatch: a new perspective. *American Ethnologist, 13*(2), 191–212.
60. Blackman, M. B. (1973). Totems to tombstones: culture change as viewed through the Haida mortuary complex, 1877-1971. *Ethnology*, *12*(1), 47–56.
61. Blackman, M. B. (1977). Ethnohistoric changes in the Haida potlatch complex. *Arctic Anthropology*, *14*(1), 39–53.
62. McIlwraith, T. F. (1948). The Bella Coola Indians (Vol. 1).Toronto: University of Toronto Press.
63. McIlwraith, T. F. (1948). The Bella Coola Indians (Vol. 2). Toronto: University of Toronto Press.
64. Elmendorf, W. W., & Kroeber, A. L. (1960). The structure of Twana culture with comparative notes on the structure of Yurok culture. *Washington State University Research Studies, 28* (3, Monographic Supp. 2), 1–576.
65. Elmendorf, W. W. (1948). The cultural setting of the Twana Secret Society. *American Anthropologist*, *50*(4), 625–633.
66. Kroeber, A. L. (1925). The Yurok. In *Handbook of the Indians of California* (pp. 1–97). Bureau of American Ethnology (Bulletin No.78). Washington, DC: Smithsonian Institute.
67. Turrado Moreno, A., & Muirden, S. (1945). *Ethnography of the Guarauno Indians*. Caracas: Vargas.
68. Olsen, D. A. (1973*). Music and shamanism of the Winikina-Warao Indians: Songs for curing and other theurgy* (Vol. 1). Ann Arbor: University Microfilms International.
69. Holmberg, A. R. (1950). *Nomads of the Long Bow: The Siriono of Eastern Bolivia*. Washington, DC: Smithsonian Institution.
70. Keane, A. H. (1884). On the Botocudos. *Journal of the Anthropological Institute of Great Britain and Ireland*, *13,* 199–213.
71. Nimuendaju, C. (1946). Social organization and beliefs of the Botocudo of Eastern Brazil. *Southwestern Journal of Anthropology, 2*(1), 93–115.
72. Henry, J. (1964). *Jungle people: a Kaingang tribe of the highlands of Brazil*. New York: Vintage.
73. Hicks, D. (1966). The Kaingang and the Aweikóma: a cultural contrast. *Anthropos, 61*(2), 839–846.
74. Métraux, A. (1947). Social organization of the Kaingang and Aweikóma according to C. Nimuendajú's unpublished data. *American Anthropologist*, *49*(1), 148–151.
75. Cooper, J. M. (1946). The Yahgan. In J. H. Stewart (Ed.), *Handbook of South American Indians, the marginal tribes* (Vol.1, pp. 81–106). Washington: U.S. Gov't.Print. Ofc.

Table A2. Hunter-gatherer populations in the study sample, their taxonomic nomenclature, representation across source trees, and proxies selected for hunter-gatherer populations absent in source trees.

Name[a]	ISO 639-3[b]	Alternative name[b]	Classification[c]	Lewis et al. (2013) classification[d]
!Kung	ktz	Ju|'hoan	Khoisan	Khoisan, Southern Africa
G/wi	gwj	|Gwi	Khoisan	Khoisan, Southern Africa
Hadza	hts	Hatsa	Khoisan	Khoisan, Hatsa
Sandawe	sad	Sandawe	Khoisan	Khoisan, Sandawe
Mbuti	efe	Efe	? (Niger-Kordofanian)	? (Nilo-Saharan)
Aka	axk	Yaka	? (Nilo-Saharan)	? (Niger-Congo)
Semang	jhi	Jehai	? (Austric, Austro-Asiatic)	? (Austro-Asiatic, Mon-Khmer)
Agta	agt	Agta	? (Austric, Austronesian)	?Austronesian, Malayo-Polynesian
Andamanese[1]	abj	Aka-Bea	Indo-Pacific, Andaman Is.	? (Andamanese)
Vedda[2,†]	ved	Veddah	? (Indo-Hittite)	? (Indo-European)
Badjau Tawi	bdl	Bajau	Austric, Austronesian	Austronesian, Malayo-Polynesian
Tiwi	tiw	Tiwi	Australian	Australian
Aranda[3,†]	axl	Aranda	Australian, Pama-Nyungan	Australian, Pama-Nyungan
Walbiri	wbp	Warlpiri	Australian, Pama-Nyungan	Australian, Pama-Nyungan
Ainu	ain	Ainu	? (Eurasiatic)	? (language isolate)
Gilyak	niv	Gilyak	? (Eurasiatic)	? (language isolate)
Yukaghir	niv	Yukaghir	? (Altaic)	? (language isolate)
Aleut	ale	Aleut	Eskimo-Aleut	Eskimo-Aleut
Copper Eskimo[4]	ikt	Inuinnaqtun	Eskimo-Aleut	Eskimo-Aleut
Montagnais	moe	Montagnais	Amerind, Almosan	Algic, Algonquian
Saulteaux[5]	ojw	Ojibwa, Western	Amerind, Almosan	Algic, Algonquian
Slave[6]	xls	Slavey, South	Na-Dene	Eyak-Athabaskan, Athabaskan
Kaska[7,†]	kkz	Kaska	Na-Dene	Eyak-Athabaskan, Athabaskan
Eyak[8,†]	eya	Eyak	Na-Dene	Eyak-Athabaskan
Haida[9]	hdn	Haida, Northern	Na-Dene	? (Haida)
Bellacoola	blc	Bella Coola	Amerind, Almosan	Salish
Twana[10,†]	twa	Twana	Amerind, Almosan	Salish
Yurok[11,†]	yur	Yurok	Amerind, Almosan	Algic
Warrau	wba	Warao	Amerind, ? (Paezan)	? (language isolate)
Siriono	srq	Siriono	Amerind, Equatorial	Tupian
Botocudo[12]	xok	Xokleng	Amerind, Macro-Ge	Jean
Aweikoma[13]	xok	Xokleng	Amerind, Macro-Ge	Jean
Yahgan	yag	Yámana	Amerind, ? (Andean)	? (language isolate)

† Population not present in source trees.

a. SCCS/eHRAF, b. *Ethnologue*, c. Greenberg and Ruhlen 2007; Ruhlen 1991, 2007, d. *Ethnologue*

1. More inclusive taxon Andamanese (oon, anq, gac) used as a proxy for Andamanese
2. Sinhalese (sin), a population speaking Sinhalese-Maldivian language related to Veddah (Lewis et al. 2013) and identified as genetically closest relative of Vedda (ref.) used as proxy for Vedda.
3. Arrernte (aer), a closely related population speaking Arandic language (Lewis et al. 2013) used as a proxy for Aranda.
4. More inclusive taxon Canadian Inuit (ikt, ike) used as a proxy for Copper Eskimo.
5. More inclusive taxon Ojibwa (ojc, ojg, ojb, ojs) used as a proxy for Saulteaux.
6. More inclusive taxon Slave (den (scs, xsl)) used as a proxy for Slave.
7. Kaska position based solely on linguistic classification
8. Eyak position based solely on linguistic classification
9. More inclusive taxon Haida (hdn, hax) used as a proxy for Haida.
10. Twana position based solely on linguistic classification.
11. Yurok position based solely on linguistic classification.
12. More inclusive taxon Kaingang (xok, zkp) used as a proxy for Botocudo.
13. More inclusive taxon Kaingang (xok, zkp) used as a proxy for Aweikoma.

Figure A1. Linguistic scaffold for supertree typology

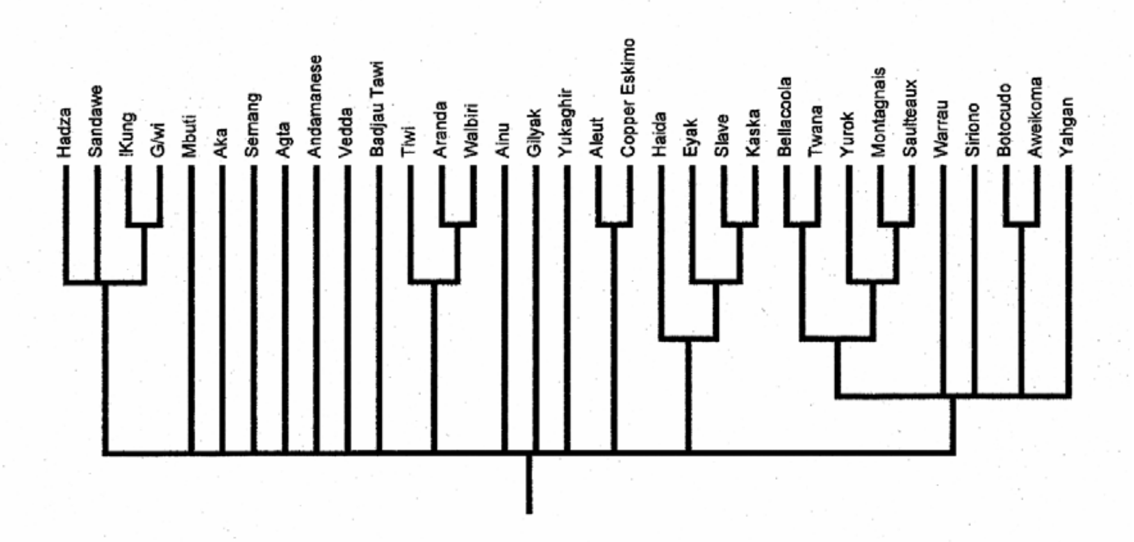

Figure A2. Supertree topology used in the study

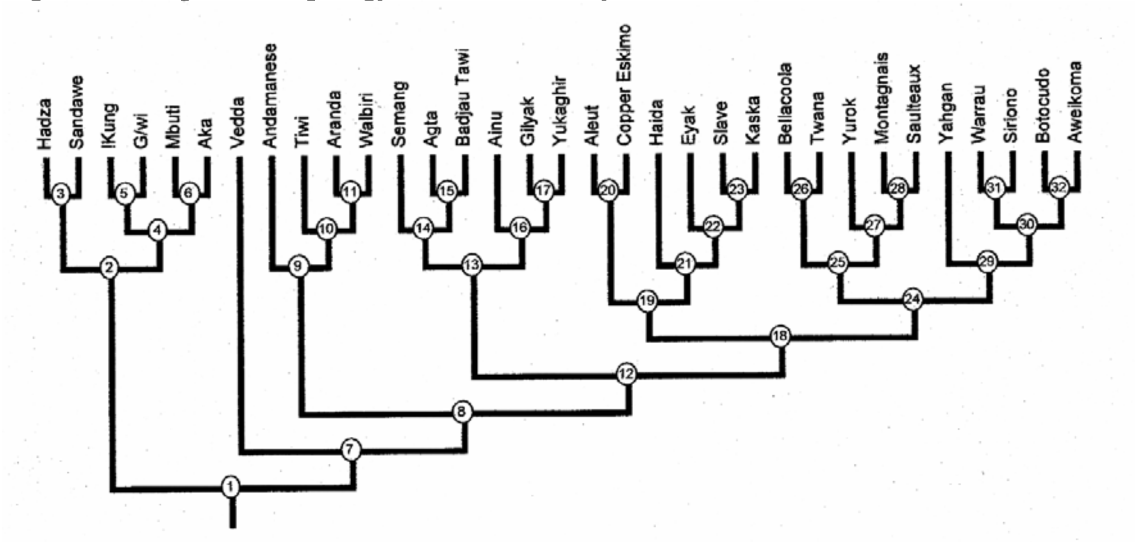

Table A3a. Divergence table of hunter-gatherer populations in the study sample

Node	Branching event	Divergence Dates
1	South African Khoisan+Pygmies+East African Khoisan divergence	An oldest divergence event in Africa
2	South African Khoisan+Pygmies/East African Khoisan divergence	
3	Hadza/Sandawe	
4	South African Khoisan/Pygmies	
5	!Kung/G/wi	North/Central South African Khoisan divergence
6	Mbuti/Aka	Eastern/Western Pygmies divergence
7	Vedda divergence	Out of Africa expansion into East Asia via ‘Southern route’
8	Andamanese + Australian Aboriginals divergence	Initial colonization of East Asia
9	Andamanese/Australian aboriginals	Initial colonization of Sahul
10	Tiwi/Aranda + Walbiri	Pama-Nyungan/non-Pama-Nyungan languages divergence
11	Aranda/Walbiri	Pama-Nyungan languages dispersal
12	East Asians/Native Americans	Colonization of East Asia via ‘Northern route’
13	South/North East Asians	
14	Semang/Philippinese	
15	Agta/Badjau Tawi	
16	Ainu/Gilyak + Yukaghir	
17	Gilyak/Yukaghir	
18	Eskimo-Aleut + Na-Dene + Amerindian divergence	Asian and Native American lineages divergence
19	Eskimo-Aleut/Na-Dene	
20	Aleut/Copper Eskimo	
21	Haida/Eyak-Athabaskan	Na-Dene languages dispersal
22	Eyak/Athabaskan	Eyak-Athabaskan languages dispersal
23	Kaska/Slave	Athabaskan languages dispersal
24	Amerindian divergence	Initial colonization of North America
25	Salish/Algic	Almosan languages dispersal
26	Bellacoola/Twana	Salishan languages dispersal
27	Yurok/Algonquian	Algic languages dispersal
28	Montagnais/Saulteaux	Algonquian languages dispersal
29	Yahgan divergence	Initial colonization of the Southern tip of South America
30	Tupi/Macro-Ge	Occupation of Lowland South America
31	Warrau/Siriono	
32	Botocudo/Aweikoma	Jean languages dispersal

Node	Time estimate (kya) - shallow divergence dates	Type of data
1	90	genetic (mtDNA, Y-chromosome, autosomal, genome-wide SNP)
2	60	genetic (mtDNA, genome-wide SNP data)
3	23	genetic (mtDNA; genome-wide SNP data)
4	51	genetic (mtDNA, genome-wide SNP data)
5	2	genetic (mtDNA)
6	**18**	genetic (mtDNA, autosomal data, genome-wide data)
7	**65**	genetic (mtDNA, autosomal data, genome-wide SNP data)
8	55.2	genetic (mtDNA)
9	50	archeological
10		genetic(mtDNA, genome-wide SNP data)
11	3 (expansion of Pama-Nyungan languages)	linguistic and archeological
12	?	
13	?	
14	30 (divergence of haplogroups in Austronesian speaking groups, Malaysia)	genetic (mtDNA + genome-wide SNP data)
15	4 (Austronesian entry into the Philippines)	archeological and linguistic
16	12 (disappearance of last land bridges between Japan/continental Asia)	archeological
17	?	
18	20	genetic (mtDNA) and archeological
19	8 (Na-Dene migration to America)	linguistic and archeological
20	5	linguistic and archeology
21	5 (appearance of coastal art, aesthetic styles associated with NW Coast)	archeological
22	3 (proto-Athabascan origin in the subarctic region of North America)	linguistic and archeological
23	2	linguistic
24	11.5 (expansion of a Beringian source population)	genetic, linguistic, archeological and paleoclimatology
25	?	
26	2 (unbroken ancestor/descendant relationships in American SW)	genetic (mtDNA)
27	4	linguistic
28	3 (dispersal of the Algonquian family)	linguistic
29	8 (disappearance of last land bridges of the Straits of Magellan)	archeological
30	6 (Macro-Ge dispersal)	linguistic
31	3 (beginning of the Tupi-Guarani expansion)	archeological
32	1.8 (expansion of Southern Jê languages through Southern Brazil)	archeological

Node	Reference
1	Knight et al 2003; Zhivotovsky et al. 2004; Gonder et al. 2007; Behar et al. 2008; Veeramah et al. 2012; Shriner et al. 2014
2	Tishkoff et al. 2007; Schlebush et al. 2012
3	Tishkoff et al. 2007
4	Tishkoff et al. 2007; Schlebush et al. 2012; Shriner et al. 2014
5	Barbieri et al. 2014
6	Chen et al. 2000; Destro-Bisol et al. 2004; Batini et al. 2007; Quintana-Murci et al. 2008; Patin et al. 2009; Verdu et al. 2009; Tishkoff et al. 2009
7	Macaulay et al. 2005; Liu et al. 2006; Gronau et al. 2011; Rasmussen et al. 2011; Fu et al. 2013b
8	Kumar et al. 2009
9	Bowler et al. 2003; O'Connell and Allen 2004; Summerhayes et al. 2010
10	Redd and Stoneking 1999; Pugach et al. 2013
11	McConvell 1996; Evans and McConvell 1998
12	
13	
14	Jinam et al. 2012
15	Pawley 2002; Reid 2013
16	Hammer et al. 2006
17	
18	Torroni et al. 1992; Saillard et al. 2000; Zlojutro et al. 2006; Goebel et al. 2008
19	Greenberg 1986
20	Greenberg 1986; Holman et al. 2011; Davis and Knecht 2010
21	Schurr et al. 2012
22	Schurr et al. 2012
23	Holman et al. 2011
24	Greenberg 1987; Kemp et al. 2007; Tamm et al. 2007; Achilli et al. 2013
25	
26	Eshleman et al. 2004
27	Golla 2007
28	Golla 2007
29	Borrero and McEwan 1997; McCulloch et al. 1997
30	Urban 1992; Callegari-Jacques et al. 2011
31	Walker et al. 2012
32	de Souza 2011

Node	Time estimate (kya) Deeper divergence dates
1	140
2	97.6
3	
4	51 (the Click Speaker ancestral component divergence)
5	35.3
6	27
7	93.5
8	87 (the Melanesian ancestral component divergence)
9	60
10	50
11	6 (expansion of Pama-Nyungan languages)
12	53 (the Native American ancestral component divergence)
13	40 (occupation of East Asia by populations ancestral to present day East Asians)
14	?
15	36 (divergence times for Australian + Papuan aboriginals + Phillippine negritos)
16	19.4 (start of spread of Y-chromosomal lineage D associated with Jomon culture)
17	?
18	30 (beginning of the radiation of Amerindian-specific mtDNA lineages)
19	12.1 (Beringian sublineage of mtDNA haplogroup A coalescence)
20	6 (Aleut-specific A and D mtDNA sublineages coalescence)
21	8 (Na-Dene portion of the HaeIII np 663 mtDNA lineage coalescence)
22	
23	2
24	16.5 (first migration from Beringia to the Americas)
25	?
26	
27	
28	
29	14.6 (earliest occupation of Mante Verde site, Chile)
30	11
31	4 (early occupation of the Orinoco river delta)
32	3 (separation of Southern Jê languages from the Northern and Central branches)

Node	Type of data
1	genetic (mtDNA; Y-chromosome, autosomal, genome-wide SNP data)
2	genetic (genome-wide SNP data)
3	genetic (mtDNA)
4	genetic (mtDNA, genome-wide SNP data)
5	genetic (mtDNA, genome-wide SNP data)
6	genetic (mtDNA)
7	genetic (genome-wide SNP data)
8	genetic (genome-wide SNP data)
9	genetic (mtDNA)
10	archeological
11	linguistic and archeological
12	genetic (genome-wide SNP data)
13	archeological and genetic (mtDNA)
14	
15	genetic (genome-wide SNP data)
16	genetic (Y-chromosome)
17	
18	genetic (mtDNA, Y-chromosome)and archeological
19	genetic (mtDNA)
20	genetic (mtDNA) and archeological
21	genetic (mtDNA)
22	linguistic
23	linguistic
24	genetic (mtDNA, Y-chromosome) and archeological
25	
26	linguistic
27	linguistic
28	linguistic
29	archeological
30	genetic and archeological
31	archeological
32	linguistic

Node	Reference
1	Chen et al. 2000; Knight et al. 2003; Zhivotovsky et al. 2004; Behar et al. 2008; Gronau et al. 2011; Veeramah et al. 2012
2	Schlebush et al. 2012
3	Gonder et al. 2007
4	Tishkoff et al. 2007; Schlebush et al. 2012; Shriner et al. 2014
5	Gonder et al. 2007, Tishkoff et al. 2007; Behar et al. 2008; Schlebush et al. 2012
6	Batini et al. 2011
7	Shriner et al. 2014
8	Shriner et al. 2014
9	Macaulay et al. 2005; Hill et al. 2006
10	O'Connell and Allen 2004
11	McConvell 1996; Evans and McConvell 1998
12	Shriner et al. 2014
13	Fu et al. 2013a
14	
15	Pugach et al. 2013
16	Hammer et al. 2006
17	
18	Torroni et al. 1992; Saillard et al. 2000; Zlojutro et al. 2006; Goebel et al. 2008
19	Achilli et al. 2013
20	Rubicz et al. 2003; Zlojutro et al. 2006; Davis and Knecht 2010
21	Torroni et al. 1992
22	Holman et al. 2011
23	Holman et al. 2011
24	Goebel et al. 2008
25	
26	Holman et al. 2011
27	Holman et al. 2011
28	Holman et al. 2011
29	Dillehay et al. 2008
30	Rothhammer and Dillehay 2009
31	Gasson 2002
32	Urban 1992; Callegari-Jacques et al. 2011

Table A3b. References for divergence dates for hunter-gatherer populations in the study sample

Achilli, A., Perego, U. A., Lancioni, H., Olivieri, A., Gandini, F., Kashani, B. H., et al. (2013). Reconciling migration models to the Americas with the variation of North American native mitogenomes. *Proceedings of the National Academy of Sciences of the United States of America, 110*(35), 14308-14313, doi:10.1073/pnas.1306290110.

Barbieri, C., Guldemann, T., Naumann, C., Gerlach, L., Berthold, F., Nakagawa, H., et al. (2014). Unraveling the Complex Maternal History of Southern African Khoisan Populations. *American Journal of Physical Anthropology, 153*(3), 435-448, doi:10.1002/ajpa.22441.

Batini, C., Coia, V., Battaggia, C., Rocha, J., Pilkington, M. M., Spedini, G., et al. (2007). Phylogeography of the human mitochondrial L1c haplogroup: Genetic signatures of the prehistory of Central Africa. [Article]. *Molecular Phylogenetics and Evolution, 43*(2), 635-644, doi:10.1016/j.ympev.2006.09.014.

Behar, D. M., Villems, R., Soodyall, H., Blue-Smith, J., Pereira, L., Metspalu, E., et al. (2008). The dawn of human matrilineal diversity. *American Journal of Human Genetics, 82*(5), 1130-1140, doi:10.1016/j.ajhg.2008.04.002.

Borrero, L. A., & McEwan, C. (1997). The peopling of Patagonia: The first human occupation. In *Patagonia. Natural History, Prehistory and Ethnography at the uttermost end of the Earth* (pp. 32-45). London: British Museum Press.

Bowler, J. M., Johnston, H., Olley, J. M., Prescott, J. R., Roberts, R. G., Shawcross, W., et al. (2003). New ages for human occupation and climatic change at Lake Mungo, Australia. *Nature, 421*(6925), 837-840, doi:10.1038/nature01383.

Callegari-Jacques, S. M., Tarazona-Santos, E. M., Gilman, R. H., Herrera, P., Cabrera, L., dos Santos, S. E. B., et al. (2011). Autosome STRs in Native South America-Testing Models of Association With Geography and Language. *American Journal of Physical Anthropology, 145*(3), 371-381, doi:10.1002/ajpa.21505.

Davis, R. S., & Knecht, R. A. (2010). Continuity and Change in the Eastern Aleutian Archaeological Sequence. *Human Biology, 82*(5-6), 507-524.

de Souza, J. G. (2011). Linguistics, archaeology, and the histories of language spread: the case of the Southern Jê languages, Brazil. *Cadernos de Etnolingüística (ISSN 1946-7095), 3*(2).

Destro-Bisol, G., Coia, V., Boschi, I., Verginelli, F., Caglià, A., Pascali, V., et al. (2004). The analysis of variation of mtDNA hypervariable region 1 suggests that Eastern and Western Pygmies diverged before the Bantu expansion. *The American Naturalist, 163*(2), 212-226.

Eshleman, J. A., Malhi, R. S., Johnson, J. R., Kaestle, F. A., Lorenz, J., & Smith, D. G. (2004). Mitochondrial DNA and prehistoric settlements: Native migrations on the western edge of North America. *Human Biology, 76*(1), 55-75, doi:10.1353/hub.2004.0019.

Evans, N., & McConvell, P. (1998). The enigma of Pama-Nyungan expansion in Australia. In R. Blench, & M. Springs (Eds.), *Archaeology and language II: Archeological Data and Linguistic Hypotheses* (pp. 174-192). London: Routledge.

Fu, Q. M., Meyer, M., Gao, X., Stenzel, U., Burbano, H. A., Kelso, J., et al. (2013). DNA analysis of an early modern human from Tianyuan Cave, China. [Article]. *Proceedings of the National Academy of Sciences of the United States of America, 110*(6), 2223-2227, doi:10.1073/pnas.1221359110.

Fu, Q. M., Mittnik, A., Johnson, P. L. F., Bos, K., Lari, M., Bollongino, R., et al. (2013). A Revised Timescale for Human Evolution Based on Ancient Mitochondrial Genomes. *Current Biology, 23*(7), 553-559, doi:10.1016/j.cub.2013.02.044.

Goebel, T., Waters, M. R., & O'Rourke, D. H. (2008). The Late Pleistocene dispersal of modern humans in the Americas. *Science, 319*(5869), 1497-1502, doi:10.1126/science.1153569.

Golla, V. (2007). Linguistic prehistory. In T. L. Jones, & K. A. Clar (Eds.), *California prehistory:*

Colonization, culture, and complexity (pp. 71-82). Plymouth: Altamira Press.
Gonder, M. K., Mortensen, H. M., Reed, F. A., de Sousa, A., & Tishkoff, S. A. (2007). Whole-mtDNA genome sequence analysis of ancient African lineages. [Article]. *Molecular Biology and Evolution, 24*(3), 757-768, doi:10.1093/molbev/msl209.
Greenberg, J. H. (1987). *Language in the Americas*: Stanford University Press.
Gronau, I., Hubisz, M. J., Gulko, B., Danko, C. G., & Siepel, A. (2011). Bayesian inference of ancient human demography from individual genome sequences. *Nature Genetics, 43*(10), 1031-U1151, doi:10.1038/ng.937.
Hammer, M. F., Karafet, T. M., Park, H., Omoto, K., Harihara, S., Stoneking, M., et al. (2006). Dual origins of the Japanese: common ground for hunter-gatherer and farmer Y chromosomes. [Article]. *Journal of Human Genetics, 51*(1), 47-58, doi:10.1007/s10038-005-0322-0.
Holman, E. W., Brown, C. H., Wichmann, S., Muller, A., Velupillai, V., Hammarstrom, H., et al. (2011). Automated Dating of the World's Language Families Based on Lexical Similarity. *Current Anthropology, 52*(6), 841-875, doi:10.1086/662127.
Chen, Y. S., Olckers, A., Schurr, T. G., Kogelnik, A. M., Huoponen, K., & Wallace, D. C. (2000). mtDNA variation in the South African Kung and Khwe - and their genetic relationships to other African populations. *American Journal of Human Genetics, 66*(4), 1362-1383, doi:10.1086/302848.
Jinam, T. A., Hong, L. C., Phipps, M. E., Stoneking, M., Ameen, M., Edo, J., et al. (2012). Evolutionary History of Continental Southeast Asians: "Early Train" Hypothesis Based on Genetic Analysis of Mitochondrial and Autosomal DNA Data. *Molecular Biology and Evolution, 29*(11), 3513-3527, doi:10.1093/molbev/mss169.
Kemp, B. M., Malhi, R. S., McDonough, J., Bolnick, D. A., Eshleman, J. A., Rickards, O., et al. (2007). Genetic analysis of early holocene skeletal remains from Alaska and its implications for the settlement of the Americas. *American Journal of Physical Anthropology, 132*(4), 605-621, doi:10.1002/ajpa.20543.
Knight, A., Underhill, P. A., Mortensen, H. M., Zhivotovsky, L. A., Lin, A. A., Henn, B. M., et al. (2003). African Y chromosome and mtDNA divergence provides insight into the history of click languages. *Current Biology, 13*(6), 464-473, doi:10.1016/s0960-9822(03)00130-1.
Kumar, S., Ravuri, R. R., Koneru, P., Urade, B. P., Sarkar, B. N., Chandrasekar, A., et al. (2009). Reconstructing Indian-Australian phylogenetic link. *Bmc Evolutionary Biology, 9*, doi:10.1186/1471-2148-9-173.
Liu, H., Prugnolle, F., Manica, A., & Balloux, F. (2006). A geographically explicit genetic model of worldwide human-settlement history. *American Journal of Human Genetics, 79*(2), 230-237, doi:10.1086/505436.
Macaulay, V., Hill, C., Achilli, A., Rengo, C., Clarke, D., Meehan, W., et al. (2005). Single, rapid coastal settlement of Asia revealed by analysis of complete mitochondrial genomes. *Science, 308*(5724), 1034-1036, doi:10.1126/science.1109792.
McConvell, P. (1996). Backtracking to Babel: the chronology of Pama-Nyungan expansion in Australia. *Archaeology in Oceania*, 125-144.
McCulloch RD, C. C., Rabassa J, Currant, AP (1997). The natural setting: The glacial and post-glacial environmental history of Fuego-Patagonia. In B. L. McEwan C, Prieto A (Ed.), *Patagonia. Natural history, prehistory and ethnography at the uttermost end of the earth* (pp. 12-31). London: British Museum Press.
O'Connell, J. F., & Allen, J. (2004). Dating the colonization of Sahul (Pleistocene Australia-New Guinea): a review of recent research. *Journal of Archaeological Science, 31*(6), 835-853, doi:10.1016/j.jas.2003.11.005.
Patin, E., Laval, G., Barreiro, L. B., Salas, A., Semino, O., Santachiara-Benerecetti, S., et al. (2009).

Inferring the Demographic History of African Farmers and Pygmy Hunter-Gatherers Using a Multilocus Resequencing Data Set. *Plos Genetics, 5*(4), doi:10.1371/journal.pgen.1000448.
Pawley, A. (2002). The Austronesian dispersal: Languages, technologies and people. In P. Bellwood, & C. Renfrew (Eds.), *Examining the Farming/Language Dispersal Hypothesis* (pp. 251-274). Cambridge: McDonald Institute for Archaeological Research.
Pugach, I., Delfin, F., Gunnarsdottir, E., Kayser, M., & Stoneking, M. (2013). Genome-wide data substantiate Holocene gene flow from India to Australia. *Proceedings of the National Academy of Sciences of the United States of America, 110*(5), 1803-1808, doi:10.1073/pnas.1211927110.
Quintana-Murci, L., Quach, H., Harmant, C., Luca, F., Massonnet, B., Patin, E., et al. (2008). Maternal traces of deep common ancestry and asymmetric gene flow between Pygmy hunter–gatherers and Bantu-speaking farmers. *Proceedings of the National Academy of Sciences, 105*(5), 1596-1601.
Rasmussen, M., Guo, X. S., Wang, Y., Lohmueller, K. E., Rasmussen, S., Albrechtsen, A., et al. (2011). An Aboriginal Australian Genome Reveals Separate Human Dispersals into Asia. *Science, 334*(6052), 94-98, doi:10.1126/science.1211177.
Redd, A. J., & Stoneking, M. (1999). Peopling of Sahul: mtDNA variation in Aboriginal Australian and Papua New Guinean populations. *American Journal of Human Genetics, 65*(3), 808-828, doi:10.1086/302533.
Reid, L. A. (2013). Who Are the Philippine Negritos? Evidence from Language. *Human Biology, 85*(1-3), 329-358.
Saillard, J., Forster, P., Lynnerup, N., Bandelt, H. J., & Norby, S. (2000). mtDNA variation among Greenland Eskimos: The edge of the Beringian expansion. *American Journal of Human Genetics, 67*(3), 718-726, doi:10.1086/303038.
Shriner, D., Tekola-Ayele, F., Adeyemo, A., & Rotimi, C. N. (2014). Genome-wide genotype and sequence-based reconstruction of the 140,000 year history of modern human ancestry. *Scientific Reports, 4*, doi:10.1038/srep06055.
Schlebusch, C. M., Skoglund, P., Sjodin, P., Gattepaille, L. M., Hernandez, D., Jay, F., et al. (2012). Genomic Variation in Seven Khoe-San Groups Reveals Adaptation and Complex African History. [Article]. *Science, 338*(6105), 374-379, doi:10.1126/science.1227721.
Schurr, T. G., Dulik, M. C., Owings, A. C., Zhadanov, S. I., Gaieski, J. B., Vilar, M. G., et al. (2012). Clan, language, and migration history has shaped genetic diversity in Haida and Tlingit populations from Southeast Alaska. *American Journal of Physical Anthropology, 148*(3), 422-435, doi:10.1002/ajpa.22068.
Summerhayes, G. R., Leavesley, M., Fairbairn, A., Mandui, H., Field, J., Ford, A., et al. (2010). Human Adaptation and Plant Use in Highland New Guinea 49,000 to 44,000 Years Ago. *Science, 330*(6000), 78-81, doi:10.1126/science.1193130.
Tamm, E., Kivisild, T., Reidla, M., Metspalu, M., Smith, D. G., Mulligan, C. J., et al. (2007). Beringian Standstill and Spread of Native American Founders. *Plos One, 2*(9), doi:10.1371/journal.pone.0000829.
Tishkoff, S. A., Gonder, M. K., Henn, B. M., Mortensen, H., Knight, A., Gignoux, C., et al. (2007). History of click-speaking Populations of Africa inferred from mtDNA and Y chromosome genetic variation. [Article]. *Molecular Biology and Evolution, 24*(10), 2180-2195, doi:10.1093/molbev/msm155.
Tishkoff, S. A., Reed, F. A., Friedlaender, F. R., Ehret, C., Ranciaro, A., Froment, A., et al. (2009). The Genetic Structure and History of Africans and African Americans. *Science, 324*(5930), 1035-1044, doi:10.1126/science.1172257.
Torroni, A., Schurr, T. G., Yang, C. C., Szathmary, E. J. E., Williams, R. C., Schanfield, M. S., et al. (1992). Native American mitochondrial DNA analysis indicates that the Amerind and the

Nadene populations were founded by two independent migrations. *Genetics*, *130*(1), 153-162.
Urban, G. (1992). A história da cultura brasileira segundo as línguas nativas. In M. Carneiro da Cunha (Ed.), *História dos Índios no Brasil* (pp. 87-102). São Paulo: Companhia das Letras.
Veeramah, K. R., Wegmann, D., Woerner, A., Mendez, F. L., Watkins, J. C., Destro-Bisol, G., et al. (2012). An Early Divergence of KhoeSan Ancestors from Those of Other Modern Humans Is Supported by an ABC-Based Analysis of Autosomal Resequencing Data. *Molecular Biology and Evolution*, *29*(2), 617-630, doi:10.1093/molbev/msr212.
Verdu, P., Austerlitz, F., Estoup, A., Vitalis, R., Georges, M., Thery, S., et al. (2009). Origins and Genetic Diversity of Pygmy Hunter-Gatherers from Western Central Africa. *Current Biology, 19*(4), 312-318, doi:10.1016/j.cub.2008.12.049.
Walker, R. S., Wichmann, S., Mailund, T., & Atkisson, C. J. (2012). Cultural Phylogenetics of the Tupi Language Family in Lowland South America. *Plos One, 7*(4), doi:10.1371/journal.pone.0035025.
Zhivotovsky, L. A., Underhill, P. A., Cinnioglu, C., Kayser, M., Morar, B., Kivisild, T., et al. (2004). The effective mutation rate at Y chromosome short tandem repeats, with application to human population-divergence time. *American Journal of Human Genetics, 74*(1), 50-61, doi:10.1086/380911.
Zlojutro, M., Rubicz, R., Devor, E. J., Spitsyn, V. A., Makarov, S. V., Wilson, K., et al. (2006). Genetic structure of the Aleuts and circumpolar populations based on mitochondrial DNA sequences: A synthesis. *American Journal of Physical Anthropology, 129*(3), 446-464, doi:10.1002/ajpa.20287.

Table A4a. Ancestral reconstruction for three characters of hunter-gatherer religiosity in all nodes: Animism, Afterlife, Shamanism

Ancestral node	Reconstruction method	Time calibration	Animism	Belief in an Afterlife	Shamanism
1	Parsimony ancestral states	-	1	0.5	0
	Likelihood ancestral states	shallow divergences	**0.98935669***	0.50525806	0.55770544
		deep divergences	**0.98855061***	0.54626414	0.57603722
2	Parsimony ancestral states	-	1	0.5	0
	Likelihood ancestral states	shallow divergences	**0.99978439***	0.49614074	0.34514788
		deep divergences	**0.9992759***	0.51593342	0.42783392
3	Parsimony ancestral states	-	1	0.5	0
	Likelihood ancestral states	shallow divergences	**0.99995378***	0.49974141	0.06760492*
		deep divergences	**0.99995883***	0.50208167	0.05310023*
4	Parsimony ancestral states	-	1	0.5	0
	Likelihood ancestral states	shallow divergences	**0.99992715***	0.49164308	0.35377935
		deep divergences	**0.99998478***	0.51717751	0.49593014
5	Parsimony ancestral states	-	1	1	1
	Likelihood ancestral states	shallow divergences	**0.99999957***	**0.99469716***	**0.99922111***
		deep divergences	**0.99998875***	0.72794605	0.81660599
6	Parsimony ancestral states	-	1	0	0
	Likelihood ancestral states	shallow divergences	**0.99997597***	0.250521	0.04693761*
		deep divergences	**0.9999904***	0.18028348	0.09180091*
7	Parsimony ancestral states	-	1	1	1
	Likelihood ancestral states	shallow divergences	**0.99961897***	0.53856939	0.83548137
		deep divergences	**0.99985014***	0.70772543	0.87487299
8	Parsimony ancestral states	-	1	1	1
	Likelihood ancestral states	shallow divergences	**0.9999881***	0.57241813	**0.91262316***
		deep divergences	**0.99998102***	0.74213059	**0.90140422***

Asterisk (*) indicates significant result $p \leq 0.05$.

Ancestral node	Reconstruction Method	Time Calibration	Animism	Belief in an Afterlife	Shamanism
9	Parsimony ancestral states	-	1	1	1
	Likelihood ancestral states	shallow divergences	**0.99996997***	0.56556156	**0.90538627***
		deep divergences	**0.99998954***	0.78460594	0.87452778
10	Parsimony ancestral states	-	1	1	1
	Likelihood ancestral states	shallow divergences	**0.99999949***	**0.99165112***	0.84383663
		deep divergences	**0.9999868***	0.8038125	0.83073051
11	Parsimony ancestral states	-	1	1	1
	Likelihood ancestral states	shallow divergences	**0.99999997***	**0.99769778***	**0.98743635***
		deep divergences	**0.99999915***	**0.99340609***	**0.99847948***
12	Parsimony ancestral states	-	1	1	1
	Likelihood ancestral states	shallow divergences	**0.99999893***	0.73089051	**0.99469604***
		deep divergences	**0.99999445***	**0.89199822***	**0.98944864***
13	Parsimony ancestral states	-	1	1	1
	Likelihood ancestral states	shallow divergences	**0.99999965***	0.73103535	**0.99593307***
		deep divergences	**0.99999992***	**0.9155599***	**0.99909357***
14	Parsimony ancestral states	-	1	1	1
	Likelihood ancestral states	shallow divergences	**0.99999485***	0.7047243	**0.99353478***
		deep divergences	**0.99999994***	**0.91351938***	**0.99911158***
15	Parsimony ancestral states	-	1	1	**1**
	Likelihood ancestral states	shallow divergences	**0.9999991***	0.53063856	**0.99931817***
		deep divergences	**0.99999752***	0.87068323	**0.99765799***
16	Parsimony ancestral states	-	1	1	1
	Likelihood ancestral states	shallow divergences	**0.9999967***	**0.91450125***	**0.9973549***
		deep divergences	**0.99999794***	**0.97538817***	**0.99837651***

Ancestral node	Reconstruction Method	Time Calibration	Animism	Belief in an Afterlife	Shamanism
17	Parsimony ancestral states	-	1	1	1
	Likelihood ancestral states	shallow divergences<br>deep divergences	**0.99999951***<br>**0.9999995***	**0.97574001***<br>**0.99381645***	**0.99948763***<br>**0.99954537***
18	Parsimony ancestral states	-	1	1	1
	Likelihood ancestral states	shallow divergences<br>deep divergences	**0.99999677***<br>**0.99999701***	0.87608822<br>**0.9633506***	**0.9969949***<br>**0.99689702***
19	Parsimony ancestral states	-	1	1	1
	Likelihood ancestral states	shallow divergences<br>deep divergences	**0.99999976***<br>**0.99999976***	**0.98712943***<br>**0.99699529***	**0.99977702***<br>**0.99977844***
20	Parsimony ancestral states	-	1	1	1
	Likelihood ancestral states	shallow divergences<br>deep divergences	**0.99999984***<br>**0.99999988***	**0.99353513***<br>**0.99899329***	**0.99986176***<br>**0.99990679***
21	Parsimony ancestral states	-	1	1	1
	Likelihood ancestral states	shallow divergences<br>deep divergences	**0.99999993***<br>**0.99999993***	**0.99353513***<br>**0.99852242***	**0.99994319***<br>**0.99993989***
22	Parsimony ancestral states	-	1	1	1
	Likelihood ancestral states	shallow divergences<br>deep divergences	**0.99999999***<br>**0.99999998***	**0.98827912***<br>**0.99557049***	**0.99998903***<br>**0.9999853***
23	Parsimony ancestral states	-	1	1	1
	Likelihood ancestral states	shallow divergences<br>deep divergences	**0.99999999***<br>**1***	**0.95388015***<br>**0.96353441***	**0.99999317***<br>**0.99999643***
24	Parsimony ancestral states	-	1	1	1
	Likelihood ancestral states	shallow divergences<br>deep divergences	**0.99999953***<br>**0.99999985***	**0.94280473***<br>**0.98661895***	**0.99861319***<br>**0.99885499***

Ancestral node	Reconstruction Method	Time Calibration	Animism	Belief in an Afterlife	Shamanism
25	Parsimony ancestral states	-	1	1	1
	Likelihood ancestral states	shallow divergences	**0.99999998***	**0.99907201***	**0.9999851***
		deep divergences	**0.99999999***	**0.99989791***	**0.99999276***
26	Parsimony ancestral states	-	1	1	1
	Likelihood ancestral states	shallow divergences	**0.99999998***	**0.99948244***	**0.99998451***
		deep divergences	**0.99999998***	**0.99987743***	**0.99998721***
27	Parsimony ancestral states	-	1	1	1
	Likelihood ancestral states	shallow divergences	**1***	**0.99959627***	**0.9999955***
		deep divergences	**1***	**0.99994808***	**0.99999633***
28	Parsimony ancestral states	-	1	1	1
	Likelihood ancestral states	shallow divergences	**0.99999998***	**0.99946611***	**0.99998532***
		deep divergences	**0.99999999***	**0.99991261***	**0.99999068***
29	Parsimony ancestral states	-	1	1	1
	Likelihood ancestral states	shallow divergences	**0.99999987***	**0.92760674***	**0.99526214***
		deep divergences	**0.99999994***	**0.98201006***	**0.99753155***
30	Parsimony ancestral states	-	1	1	1
	Likelihood ancestral states	shallow divergences	**0.99999994***	0.86947973	**0.97269425***
		deep divergences	**0.99999989***	**0.93207902***	**0.97216753***
31	Parsimony ancestral states	-	1	1	1
	Likelihood ancestral states	shallow divergences	**0.99999994***	0.79676298	**0.94075587***
		deep divergences	**0.99999994***	0.84931385	**0.9264907***
32	Parsimony ancestral states	-	1	1	1
	Likelihood ancestral states	shallow divergences	**0.99999997***	0.77185503	**0.92859314***
		deep divergences	**0.99999996***	0.83886236	**0.92033659***

Table A4b. Ancestral reconstruction for four characters of hunter-gatherer religiosity in all nodes: Ancestor Worship, High Gods, Active High Gods and Ancestor Worship

Ancestral node	Reconstruction Method	Time calibration	Ancestor Worship	High Gods	Active High Gods	Active Ancestor Worship
1	Parsimony	-	0	0	0	0
	Likelihood Ancestral states	shallow divergences	0.5	0.5	0.04649112*	0.43429948
		deep divergences	0.5	0.5	0.07916184*	0.10739427*
2	Parsimony	-	0	0	0	0
	Likelihood ancestral states	shallow divergences	0.49999984	0.5	0.06306996*	0.3534881
		deep divergences	0.5	0.49999999	0.12071412	0.06484999*
3	Parsimony	-	0	0	0	0
	Likelihood ancestral states	shallow divergences	0.5	0.49967258	0.01172006*	0.18041384
		deep divergences	0.5	0.49676052	0.01412709*	0.01828653*
4	Parsimony	-	0	0	0	0
	Likelihood ancestral states	shallow divergences	0.49999815	0.5	0.09924286*	0.33153467
		deep divergences	0.5	0.5	0.29764904	0.01194196*
5	Parsimony	-	0	1	1	0
	Likelihood ancestral states	shallow divergences	0.06488181*	0.89889098*	**0.99946059***	0.00201257*
		deep divergences	0.5	0.50133933	0.80457262	0.00837643*
6	Parsimony	-	0	0	0	0
	Likelihood ancestral states	shallow divergence	0.49221734	0.49812636	0.00958869*	0.12433432
		deep divergences	0.49999989	0.4936424	0.03991104*	0.00594483*
7	Parsimony	-	0.5	0	0	0
	Likelihood Ancestral states	shallow divergences	0.50000002	0.5	0.0147429*	0.40874244
		deep divergences	0.5	0.49999995	0.01351448*	0.08523942*
8	Parsimony	-	0.5	0	0	0
	Likelihood ancestral states	shallow divergences	0.50000011	0.49999999	0.00509984*	0.34535215
		deep divergences	0.5	0.49999988	0.00723788*	0.05094172*

Ancestral node	Reconstruction Method	Time Calibration	Ancestor Worship	High Gods	Active High Gods	Active Ancestor Worship
9	Parsimony	-	0.5	0	0	0
	Likelihood ancestral states	shallow divergences	0.50000086	0.49999997	0.00751864*	0.33788734
		deep divergences	0.5	0.49998812	0.00506884*	0.01994129*
10	Parsimony	-	0.5	0	0	0
	Likelihood ancestral states	shallow divergences	0.86638714	0.4003842	0.00008568*	0.002961*
		deep divergences	0.5	0.49996439	0.00511664*	0.01621562*
11	Parsimony	-	0.5	0	0	0
	Likelihood ancestral states	shallow divergences	**0.91601139***	0.23857514	0.00000794*	0.00056653*
		deep divergences	0.52822414	0.20606209	0.00020569*	0.00045198*
12	Parsimony	-	0.5	0	0	0
	Likelihood ancestral states	shallow divergences	0.4999709	0.49999631	0.00269126*	0.2670984
		deep divergences	0.5	0.4999766	0.0022686*	0.01833662*
13	Parsimony	-	0.5	0.5	0	0
	Likelihood ancestral states	shallow divergences	0.49998523	0.50000421	0.00385904*	0.27570418
		deep divergences	0.5	0.50053666	0.00237663*	0.02320494*
14	Parsimony	-	0	0.5	0	0
	Likelihood ancestral states	shallow divergences	0.49965116	0.50001396	0.02166731*	0.24326767
		deep divergences	0.5	0.50053968	0.00285221*	0.02030816*
15	Parsimony	-	0	0.5	0	0
	Likelihood ancestral states	shallow divergences	0.19529316	0.5	0.00023072*	0.00718567*
		deep divergences	0.5	0.50027495	0.00235248*	0.01497359*
16	Parsimony	-	1	0.5	0	1
	Likelihood ancestral states	shallow divergences	0.55725585	0.50737492	0.00062315*	**0.93293674***
		deep divergences	0.50001471	0.51254573	0.00058618*	**0.94440009***

Ancestral node	Reconstruction Method	Time Calibration	Ancestor Worship	High Gods	Active High Gods	Active Ancestor Worship
17	Parsimony	-	1	1	0	1
	Likelihood ancestral states	shallow divergences	0.69400272	0.62055337	0.00009844*	**0.98948634***
		deep divergences	0.50313132	0.65638219	0.00013518*	**0.99662563***
18	Parsimony	-	0.5	0	0	0
	Likelihood ancestral states	shallow divergences	0.49826689	0.49850052	0.00103311*	0.15734521
		deep divergences	0.49999998	0.49472749	0.00124285*	0.01505229
19	Parsimony	-	0.5	0	0	0
	Likelihood ancestral states	shallow divergences	0.45586828	0.44045262	0.00004553*	0.28690215
		deep divergences	0.49932472	0.40121434	0.00006578*	0.0820227*
20	Parsimony	-	1	0	0	1
	Likelihood ancestral states	shallow divergences	0.68882479	0.47909209	0.00002802*	**0.88555905***
		deep divergences	0.52818629	0.46849228	0.00002801*	**0.96743862***
21	Parsimony	-	0	0	0	0
	Likelihood ancestral states	shallow divergences	0.19239451	0.33042693	0.00001137*	0.02137913*
		deep divergences	0.48374344	0.28716776	0.00001737*	0.00370175*
22	Parsimony	-	0	0	0	0
	Likelihood ancestral states	shallow divergences	0.05982445	0.2874466	0.00000222*	0.00085507*
		deep divergences	0.38447096	0.23094688	0.00000443*	0.00006444*
23	Parsimony	-	0.5	0	0	0
	Likelihood ancestral states	shallow divergences	0.02906697	0.35004854	0.00000142*	0.0001209*
		deep divergences	0.21364036	0.32182248	0.00000109*	0.00000285*
24	Parsimony	-	0.5	0	0	0
	Likelihood ancestral states	shallow divergences	0.49982502	0.48841483	0.0027186*	0.03498002*
		deep divergences	0.5	0.47689201	0.00319949*	0.00165965*

Ancestral node	Reconstruction Method	Time Calibration	Ancestor Worship	High Gods	Active High Gods	Active Ancestor Worship
25	Parsimony	-	0.5	0	0	0
	Likelihood ancestral states	shallow divergences	0.35041263	0.23064584	0.0000053*	0.00025072*
		deep divergences	0.48629034	0.15911451	0.0000039*	0.00000516*
26	Parsimony	-	0.5	0	0	0
	Likelihood ancestral states	shallow divergences	0.42170835	0.38340443	0.00000333*	0.00012641*
		deep divergences	0.49640135	0.27635687	0.00000399*	0.00000806*
27	Parsimony	-	0.5	0	0	0
	Likelihood ancestral states	shallow divergences	0.3326565	0.19004611	0.00000095*	0.00007796*
		deep divergences	0.48098824	0.12137504	0.0000012*	0.0000023*
28	Parsimony	-	0.5	0	0	0
	Likelihood ancestral states	shallow divergences	0.37222357	0.13970302	0.00000312*	0.00012309*
		deep divergences	0.49485905	0.05293082	0.00000288*	0.00000587*
29	Parsimony	-	0.5	0.5	0	0
	Likelihood ancestral states	shallow divergences	0.54906057	0.53114357	0.02124681*	0.03821882*
		deep divergences	0.50007616	0.50257617	0.01054609*	0.00253994*
30	Parsimony ancestral states	-	0.5	0.5	0	0
	Likelihood ancestral states	shallow divergences	0.52860332	0.5263287	0.01481972*	0.0788802*
		deep divergences	0.50000896	0.50746724	0.00797786*	0.02437226*
31	Parsimony ancestral states	-	0.5	1	0	0
	Likelihood ancestral states	shallow divergences	0.51273333	0.79383241	0.03472997*	0.13325871
		deep divergences	0.50000014	0.87539264	0.04073876*	0.06411901*
32	Parsimony ancestral states	-	0.5	0	0	0
	Likelihood ancestral states	shallow divergences	0.50921197	0.09371939*	0.00008399*	0.15299041
		deep divergences	0.50000008	0.07598965*	0.00005246*	0.06951924*

Table A5. Pagel's test for correlated discrete character evolution

Character X	Character Y		Independent model	Dependent model
Animism	Belief in an Afterlife	q12(alpha1)	0.017922678171600308	2.955006068003479E-7
		q13(alpha2)	3.5141401263485466	3.876039800319336E-6
		q21(beta1)	7.547566959924306E-4	4.876833291438565
		q31(beta2)	1.0812738648986855	0.001330532761444173
		q24	-	6.963364043052747
		q34	-	11.770106926545987
		q42	-	1.916250063082768E-5
		q43	-	2.753450428794025
		**log Likelihood (L)**	-20.611133199198772	-18.5037576532465
		**log Likelihood difference (LD)**	2.107375545952273	
		**p-value**	**0.041000000000000036***	
Animism	Shamanism	q12(alpha1)	0.017922679679795207	1.0453676822495886E-4
		q13(alpha2)	2.611746557985332	5.466212448651201E-7
		q21(beta1)	7.547530435731249E-4	3.2146092549083307
		q31(beta2)	0.8036310168494801	9.801534639612092E-4
		q24	-	2.986507679741015
		q34	-	2.3082172206318075
		q42	-	3.878578851844866E-5
		q43	-	0.6022309534477134
		**log Likelihood (L)**	-20.61113662702108	-18.43901665082429
		**log Likelihood difference (LD)**	2.1721199761967895	
		**p-value**	**0.029000000000000026***	
Animism	High Gods	q12(alpha1)	0.01792268529659327	1.0615647497622018E-5
		q13(alpha2)	0.16814429612889825	5.003935597094861E-7
		q21(beta1)	7.547560491754113E-4	0.9826259441965896
		q31(beta2)	0.2728853651313188	2.4115585431978422E-4
		q24	-	0.9668616658801443
		q34	-	0.4816886228320609
		q42	-	2.5009443596585324E-5
		q43	-	0.7121275394333404
		**log Likelihood (L)**	-24.58771065166488	-23.506430016941867
		**log Likelihood difference (LD)**	1.081280634723015	
		**p-value**	0.17900000000000005	

Character X	Character Y		Independent model	Dependent model
Belef in an Afterlife	Shamanism	q12(alpha1)	5.218879100103742	3.769966946383435E-5
		q13(alpha2)	9.1998518117755	0.6567008570002618
		q21(beta1)	1.6058090787256674	1.2789864728129487
		q31(beta2)	2.8307237237801117	0.7640944397067033
		q24	-	1.275241216945288
		q34	-	1.1884735867468457
		q42	-	0.21069875170004448
		q43	-	4.1732939719157696E-7
		**log Likelihood (L)**	-35.71413666880457	-28.747538310792255
		**log Likelihood difference (LD)**	6.966598358012316	
		**p-value**	**0.0***	
Belef in an Afterlife	Ancestor Worship	q12(alpha1)	4.062588573214016	0.07729929525467856
		q13(alpha2)	0.1316880632191157	0.3423011923963714
		q21(beta1)	1.2500272153269685	9.849092744435119
		q31(beta2)	0.15935550941025572	0.11805877538869874
		q24	-	1.859174871172054
		q34	-	0.13335933543247983
		q42	-	0.11193154654817793
		q43	-	9.717776444793614E-8
		**log Likelihood (L)**	-40.48177159569678	-34.65735223063223
		**log Likelihood difference (LD)**	5.82441936506455	
		**p-value**	**0.0030000000000000027***	
Belef in an Afterlife	High Gods	q12(alpha1)	4.4941435818152	1.5833328218838048
		q13(alpha2)	0.16814436626199697	2.428529741694923
		q21(beta1)	1.3828132507988233	3.1337798343938976
		q31(beta2)	0.2728854530140183	0.6503667749932317
		q24	-	7.829647431342137E-6
		q34	-	0.11160233996231926
		q42	-	0.17082008637351465
		q43	-	3.035481021877122E-8
		**log Likelihood (L)**	-39.69071419127651	-39.337955277123875
		**log Likelihood difference (LD)**	0.3527589141526377	
		**p-value**	0.736	

Character X	Character Y		Independent model	Dependent model
Shamanism	Ancestor Worship	q12(alpha1)	4.395323925248568	41.625709147923466
		q13(alpha2)	0.13168786236526236	0.13779603960001932
		q21(beta1)	1.3524075349737754	101.14497024117185
		q31(beta2)	0.15935523841411559	2.919637565200422E-7
		q24	-	0.5929928616459182
		q34	-	0.08712683229206851
		q42	-	0.1676741087519177
		q43	-	7.131724941334274E-7
		**log Likelihood (L)**	-40.48177159146289	-36.602667272039206
		**log Likelihood difference (LD)**	3.879104319423682	
		**p-value**	**0.010000000000000009***	
Shamanism	Active Ancestor Worship	q12(alpha1)	4.254852997372292	0.21941655975829275
		q13(alpha2)	0.024119413532695626	1.7896916645488917E-5
		q21(beta1)	1.3091859333853737	3.6826969861781986
		q31(beta2)	0.07192919182033762	0.03730735853886139
		q24	-	11.81473513324769
		q34	-	1.7747220847551834E-6
		q42	-	0.4163554402784801
		q43	-	0.059289818102207396
		**log Likelihood (L)**	-34.73547880446629	-28.717411054843915
		**log Likelihood difference (LD)**	6.018067749622375	
		**p-value**	**0.0010000000000000009***	
Ancestor Worship	High Gods	q12(alpha1)	0.13168795974909628	0.12272137671597863
		q13(alpha2)	0.16814428896352349	0.34750012782276024
		q21(beta1)	0.1593553811356608	0.2770546994789072
		q31(beta2)	0.2728853524995929	0.5496547163764938
		q24	-	6.226891899527741E-6
		q34	-	5.4257204773097305
		q42	-	2.236504233470032E-5
		q43	-	6.214980189961206
		**log Likelihood (L)**	-44.45834911234516	-43.560892189874494
		**log Likelihood difference (LD)**	0.897456922470667	
		**p-value**	0.529	

Character X	Character Y		Independent model	Dependent model
Ancestor Worship	Active Ancestor Worship	q12(alpha1)	0.13168794114432877	0.04236040487972608
		q13(alpha2)	0.024119429548873753	0.04301070470687921
		q21(beta1)	0.15935535472867446	7.93553170358179
		q31(beta2)	0.07192921083907078	0.12185613637053422
		q24	-	15.919166612679389
		q34	-	1.0147909229550855E-5
		q42	-	0.17943526424508463
		q43	-	4.3823101954721057E-7
		**log Likelihood (L)**	-39.5031137244075	-30.182930342847683
		**log Likelihood difference (LD)**	9.320183381559815	
		**p-value**	**0.0***	
High Gods	Active High Gods	q12(alpha1)	0.1681442651727595	3.642066233186282E-7
		q13(alpha2)	0.018183457243443422	0.25937024676865095
		q21(beta1)	0.2728853051596625	14.92329103446345
		q31(beta2)	0.09893275509479003	0.6885715270306392
		q24	-	3.919324295428966
		q34	-	0.06872501587988517
		q42	-	1.6855097188720912E-6
		q43	-	0.1061500550737619
		**log Likelihood (L)**	-33.579043578951854	-27.93444889583701
		**log Likelihood difference (LD)**	5.644594683114843	
		**p-value**	**0.0010000000000000009***	
Active High Gods	Active Ancestor Worship	q12(alpha1)	0.018183458135025643	0.03218896757368417
		q13(alpha2)	0.024119427628359044	0.00624367320658855
		q21(beta1)	0.09893274533510152	0.07723439624163879
		q31(beta2)	0.07192920929065762	0.04597475587572726
		q24	-	7.7471500624074565
		q34	-	6.597699131357802E-7
		q42	-	54.09955636099969
		q43	-	0.11583308097744709
		**log Likelihood (L)**	-28.623808191014163	-27.726982419315043
		**log Likelihood difference (LD)**	0.8968257716991204	
		**p-value**	0.5860000000000001	
